# Prevalence and factors associated with psychological burden in COVID-19 patients and their relatives: A prospective observational cohort study

**DOI:** 10.1371/journal.pone.0250590

**Published:** 2021-05-05

**Authors:** Katharina Beck, Alessia Vincent, Christoph Becker, Annalena Keller, Hasret Cam, Rainer Schaefert, Thomas Reinhardt, Raoul Sutter, Kai Tisljar, Stefano Bassetti, Philipp Schuetz, Sabina Hunziker

**Affiliations:** 1 Medical Communication and Psychosomatic Medicine, University Hospital Basel, Basel, Switzerland; 2 Division of Clinical Psychology and Psychotherapy, Faculty of Psychology, University of Basel, Basel, Switzerland; 3 Emergency Department, University Hospital Basel, Basel, Switzerland; 4 Department for Psychosomatic Medicine, University Hospital Basel, Basel, Switzerland; 5 Medical Faculty of the University of Basel, Basel, Switzerland; 6 Human Resources & Leadership Development, University Hospital Basel, Basel, Switzerland; 7 Intensive Care Unit, University Hospital Basel, Basel, Switzerland; 8 Department of Clinical Research, University Hospital Basel, Basel, Switzerland; 9 Division of Internal Medicine, University Hospital Basel, Basel, Switzerland; 10 Division of Internal Medicine, Kantonsspital Aarau, Aarau, Switzerland; CentERdata, NETHERLANDS

## Abstract

**Background:**

Due to the dramatic measures accompanying isolation and the general uncertainty and fear associated with COVID-19, patients and relatives may be at high risk for adverse psychological outcomes. Until now there has been limited research focusing on the prevalence of psychological distress and associated factors in COVID-19 patients and their relatives. The objective of our study was to assess psychological distress in COVID-19 patients and their relatives 30 days after hospital discharge.

**Methods:**

In this prospective observational cohort study at two Swiss tertiary-care hospitals we included consecutive adult patients hospitalized between March and June 2020 for a proven COVID-19 and their relatives. Psychological distress was defined as symptoms of anxiety and/or depression measured with the Hospital Anxiety and Depression Scale (HADS), i.e., a score of ≥8 on the depression and/or anxiety subscale. We further evaluated symptoms of post-traumatic stress disorder (PTSD), defined as a score of ≥1.5 on the Impact of Event Scale-Revised (IES-R).

**Results:**

Among 126 included patients, 24 (19.1%) had psychological distress and 10 (8.7%) had symptoms of PTSD 30 days after hospital discharge. In multivariate logistic regression analyses three factors were independently associated with psychological distress in patients: resilience (OR 0.82; 95%CI 0.71 to 0.94; p = 0.005), high levels of perceived stress (OR 1.21; 95%CI 1.06 to 1.38; p = 0.006) and low frequency of contact with relatives (OR 7.67; 95%CI 1.42 to 41.58; p = 0.018). The model showed good discrimination, with an area under the receiver-operating characteristic curve (AUC) of 0.92. Among 153 relatives, 35 (22.9%) showed symptoms of psychological distress, and 3 (2%) of PTSD. For relatives, resilience was negatively associated (OR 0.85; 95%CI 0.75 to 0.96; p = 0.007), whereas perceived overall burden caused by COVID-19 was positively associated with psychological distress (OR 1.72; 95%CI 1.31 to 2.25; p<0.001). The overall model also had good discrimination, with an AUC of 0.87.

**Conclusion:**

A relevant number of COVID-19 patients as well as their relatives exhibited psychological distress 30 days after hospital discharge. These results might aid in development of strategies to prevent psychological distress in COVID-19 patients and their relatives.

## Introduction

In December 2019, a novel Coronavirus causing the Coronavirus disease 2019 (COVID-19) emerged in Wuhan, China, leading to a global pandemic. The clinical symptoms of COVID-19 range from mild flu-like symptoms to acute respiratory distress syndrome [1, 2]. While children and healthy young adults are often less affected by the disease, vulnerable individuals such as the elderly and people with chronic lung disease or cardiovascular comorbidities are at high risk of experiencing complicated courses needing invasive ventilation or circulatory support [3, 4].

Recent studies suggest that COVID-19 causes a relevant increase in risks of mortality and morbidity [5–7]. Although the true impact of COVID-19 on mortality and morbidity has become more evident in recent studies, insights regarding psychological burden beyond the acute phase of the illness in these patients and their relatives who may be at high risk for adverse psychological outcomes is limited [8–11]. In fact, most countries, including Switzerland, have implemented orders to isolate at home or other quarantine measures to contain the spread of COVID-19. As a consequence, patients hospitalized for COVID-19 are often quarantined, and visits—also by family members—are limited to prevent further spread of the virus. Research during previous epidemics showed that these may be associated with adverse psychological effects on patients and relatives, including an increased risk of anxiety disorders, depression and post-traumatic stress disorder [PTSD; 8, 11, 12, 13–17]. Research on the short-term psychological consequences of the COVID-19 pandemic has shown adverse psychological effects [18–20]. For instance, a large Swiss survey including 10472 participants of the general public found the prevalence of moderately severe or severe depressive symptoms to increase from 9.1% during confinement at the time of the first pandemic wave to 11.7% during the following partial confinement, and 18% during the second wave [21, 22]. When asked about their symptom levels before the pandemic, i.e., during the first two weeks of February 2020, only 3.4% of participants reported moderately severe or severe depressive symptoms. A cross-sectional German study evaluating 15037 participants from the general population during the beginning of the pandemic reported rates of depressive and anxiety symptoms of 14.3% and 19.7%, respectively [23]. Retrospectively assessed rates of depressive and anxiety symptoms before the pandemic were significantly lower with rates of 7.6% and 9%, respectively. While including large samples, interpretation of findings of these studies is partially limited due to their naturalistic approach and lack of pre-COVID-19 data. Findings of prospective studies assessing probability samples of the general population yielded mixed results. Two prospective studies analyzing the prevalence of anxiety [24] and depression [24, 25] before and after the outbreak in two different samples of the general population each, found an increase in clinically relevant symptoms. Contrary, a Dutch long-term study assessing prevalence of moderate to high levels of anxiety or depression in the general population in November 2019 and March 2020 did not show an increase with rates being 16.9% and 17.0%, respectively [26] and a later follow-up assessment in June 2020 even revealed a significant decrease to 15.3% [27]. Findings of a similar Dutch long-term study in older adults and a study comparing serious psychological distress in two samples of the US general population were in line with this [28, 29].

Insight regarding psychological distress of patients with COVID-19 is limited, so far. A meta-analysis including 50 mostly Chinese studies on the general population, healthcare workers and patients with COVID-19 showed a pooled prevalence of 44% with psychological morbidities [9]. Four of the included studies had assessed patients with COVID-19, yielding a pooled prevalence of 42% for depression, 37% for anxiety disorders and 96% for post-traumatic stress symptoms. The findings regarding depression and anxiety are in line with other meta-analyses and systematic reviews on various populations, few of them patient samples, affected by the COVID-19 pandemic [10, 30] and more recent studies on hospitalized patients. Regarding post-traumatic stress symptoms, a meta-analysis including more recent studies than the meta-analysis of Krishnamoorthy et al. [9] yielded a pooled prevalence of 24% of post-traumatic stress symptoms [31]. Still, studies on samples of the general population included in these systematic reviews and meta-analyses should be viewed with caution due to methodological issues including low representativeness and other sources of bias.

Relatives of patients hospitalized with COVID-19 might be equally affected but evidence is scarce. The study of Dorman-Ilan et al. [32] suggests that both isolated COVID-19 patients and relatives might suffer from similarly high levels of anxiety and depressive symptoms during the initial stage of hospitalization.

While heightened psychological distress during the acute phase of the illness in patients and their relatives can be expected, it might be additionally relevant to investigate how many experience clinically relevant symptoms persisting beyond that initial phase and which characteristics might be related to this. However, only few studies evaluated this, so far. Recent studies from Italy, Turkey and China investigating COVID-19 survivors about one to two months after hospital discharge found a prevalence of 10% to 42% for anxiety [33–35], 11% to 31% for depression [33–35], 12% to 28% for PTSD [33, 35, 36], and 40% for insomnia [33], suggesting persisting psychological distress in a considerable number of patients. Furthermore, a recent Chinese study revealed that 23% of patients still experienced anxiety or depression even 6 months after discharge [37].

Factors associated with increased psychological distress might include sociodemographic, illness-related, psychosocial and hospital-related characteristics [8, 11]. A systematic review on the psychological impact of past viral respiratory epidemics indicated that female patients and those with lower education levels experience increased anxiety, depression and PTSD [8]. Studies evaluating psychological distress in the context of COVID-19 found female gender [32, 37–40], higher age [39, 40], lower education level [39] and not being employed [40] to be associated with anxiety. Further, female gender [37, 38, 40], lower education [18, 35], not being employed [40] and living with children [35] were potential risk factors for depression. Regarding symptoms of PTSD, female gender [36, 41], younger age [41] and not being employed [36] emerged as potential risk factors. Also, previous research shows that people who follow disaster media closely have higher levels of post-traumatic stress symptoms and psychological distress [42].

Similar to studies on clinical conditions such as traffic accidents [43], stroke [44] or cardiac arrest [45, 46] which found considerable rates of PTSD symptoms, anxiety and depression, psychological distress in COVID-19 patients and relatives might be related to the potentially life-threatening illness requiring hospitalization or critical care and uncertainty about the course or outcome [11, 47–49]. In line with this, duration of hospitalization [40], higher disease severity [35, 37] and ICU stay [11, 50] might be associated with increased psychological distress.

A recent review on the effects of quarantine measures during past outbreaks suggests a negative impact on psychological well-being of patients as well as their relatives especially due to separation from partners and relatives [12]. However, these findings are difficult to transfer as previous outbreaks were either localized or limited in time and by far did not reach the extent of the current COVID-19 pandemic. Studies during the COVID-19 pandemic found perceived stigmatization and feeling isolated with inadequate social support to be associated with increased anxiety, depression and symptoms of PTSD [18, 35, 36]. Lockdown measures may also lead to financial and occupational concerns and contribute to psychological distress [18, 41, 51]. A large study evaluated the association of internal coping mechanisms for emotion regulation with anxiety, depression and symptoms of PTSD applying a machine learning model in 2787 individuals of the general population. Low use of adaptive defense mechanisms, e.g., humor and self-assertion to regulate one’s emotions was associated with heightened levels of anxiety, depression and symptoms of PTSD [39]. In the context of potentially protective coping mechanisms, resilience, often defined as the ability to successfully cope with adverse life events, might also be related to psychological distress [52] and is potentially modifiable [53]. A meta-analysis including longitudinal as well as cross-sectional studies evaluating correlations between resilience and mental health showed that resilience is negatively correlated to negative indicators of mental health, such as depression, anxiety and negative affect, and positively correlated to positive indicators of mental health, such as life satisfaction and positive affect [52]. Further, a review on the role of resilience as a protective factor regarding anxiety, depression and post-traumatic stress during the COVID-19 pandemic revealed that “resilient” coping strategies to deal with COVID-19-related distress are common [53]. However, evidence on the nature of the association of resilience and psychological distress is still inconclusive [54–56] and more research is needed to identify effective interventions [53]. Dorman-Ilan et al. [32] found that relatives who did not feel protected by the hospital might suffer from increased anxiety even one month after patients’ discharge.

Though there is growing evidence on acute psychological distress in the context of COVID-19, evidence on prevalence and factors associated with persisting psychological distress in patients and their relatives is scarce. Herein, our aim was to assess in parallel the prevalence of and factors associated with persisting psychological burden in COVID-19 patients and their relatives one month after hospital discharge. Such insights may help to prevent these adverse outcomes by focusing on modifiable risk factors and identifying specific treatments to support patients and relatives in the near future.

## Materials and methods

### Study setting

We conducted this prospective observational cohort study at two tertiary care hospitals in Switzerland—the University Hospital Basel and the Kantonsspital Aarau—from March until June 2020. The study was approved by the local Ethics Committee (Ethics Committee Northwest and Central Switzerland EKNZ, approval reference number: 2019–01162). All participating patients and relatives provided written informed consent. This manuscript adheres to the STROBE statement [57; see S1 File].

### Study population

We screened all consecutively admitted COVID-19 patients and their closest relatives upon hospitalization regarding inclusion and exclusion criteria. COVID-19 was confirmed by reverse transcriptase polymerase chain reaction from nasopharyngeal swabs [45, 58]. Relatives were chosen according to surrogate decision-making rank (spouse > parents/adult children > others) as indicated in patients’ medical records. Exclusion criteria for patients and relatives were insufficient knowledge of the local language (German), cognitive impairment, i.e., a condition where patients were not able to understand and respond to the questions of our interview including dementia, delirium and others, or serious psychiatric conditions, e.g., psychosis. Relatives who were subsequently hospitalized due to COVID-19 were included in the patient sample only. There were no exclusions based on patient characteristics and severity or duration of COVID-19 disease. We contacted relatives during hospitalization and patients about one month after hospital discharge by phone and invited them to participate in our study. Those who had agreed received a letter including the study information and informed consent form which they were asked to sign and return. Relatives and patients were excluded if no informed consent was provided.

### Collection of potential predictor and outcome variables of patients and relatives

In this prospective observational cohort study, we conducted telephone interviews with all participating patients and relatives one month after hospital discharge to collect data on potential risk and protective factors concerning the time of hospitalization as well as on psychological outcome at the time of the assessment. For patients we additionally reviewed their medical charts to obtain relevant medical information. For relatives of patients that were hospitalized during the study period, we did a baseline interview upon admission of the patient. Several predictor variables specific to COVID-19 were assessed by items specifically designed for the purpose of this study. For the assessment of the other factors, we used well-established clinical risk scores and validated psychometric measures. We assessed potential predictor variables from four domains, i.e., sociodemographic, illness-related, psychosocial and hospital-related factors. While items in the sociodemographic domain were the same for both patients and relatives, factors in the other three domains partially differed to account for patient- and relative-specific characteristics (see Tables 2 and 3).

### Variables collected upon hospitalization

Sociodemographic factors were assessed for patients and relatives and included age, gender, citizenship, cultural background, religious affiliation, civil status, children and current job situation.

#### Illness-related factors

For patients, in the domain of illness-related factors we assessed variables such as timepoint of COVID-19 diagnosis, duration of hospitalization, antibiotics during hospitalization, investigational therapy, anxiolytics during hospitalization, ICU stay, and intubation. Based on patients’ medical condition at the end of their hospitalization for COVID-19, we calculated the Charlson Comorbidity Index (CCI) [59], a score which characterizes the severity of comorbidity and predicts ten-year mortality. Further, we collected patients’ vital signs and calculated the National Early Warning Score (NEWS) [60], a commonly used tool that assesses the severity of a patient’s illness and detects patients prone to clinical deterioration.

For relatives, the domain of illness-related factors included items assessing if the relative was quarantined or infected with SARS CoV-2, the time point of the patient’s COVID-19 diagnosis, and if the patient had died due to COVID-19.

#### Psychosocial factors

For relatives, the relationship with patient and whether they lived in the same household as the patients was assessed.

### Variables collected at 30 days after hospital discharge

#### Illness-related factors

Self-perceived overall health status was assessed using the visual analogue scale (VAS) of the EuroQol, ranging from 0 (worst imaginable health) to 100 (best imaginable health) at 30-day follow-up for patients and relatives [61, 62].

#### Psychosocial factors

For both patients and relatives, psychosocial factors were assessed, such as pre-existing psychological comorbidities, and intake of psychotropic drugs, the amount of COVID-19 media consumption and worries due to COVID-19 media reports (on a VAS 0–10), the frequency of contact between patients and relatives, as well as type of communication. Patients’ and relatives’ pre-existing psychological comorbidities were inquired during the telephone interview by asking participants directly if psychological comorbidities had been diagnosed previously, e.g., depression, anxiety disorder as well as through questions about psychotherapeutic or pharmaceutic treatment, e.g., antidepressants. In patients, we additionally reviewed medical charts regarding information on pre-existing psychological comorbidities. Further, items designed for the purpose of this study were assessed, such as current worries or burdens and helpfulness of different coping strategies, all rated on a VAS 0–10.

Also, we evaluated perceived stress of patients and relatives with the Perceived Stress Scale (10-item version; PSS-10; Cronbach’s alphas ≥0.80), a well-established self-report measure assessing how unpredictable, uncontrollable and overloaded respondents perceived their life during the last month [63, 64]. Further, we estimated resilience of patients and relatives using the 10-item version of the Connor-Davidson Resilience Scale (CD-RISC-10), which refers to the preceding month and assesses characteristics of resilience that can also be framed as stress-coping ability [65]. The CD-RISC is widely applied in clinical research and the original 25-item questionnaire as well as the 10-item version showed good validity with a Cronbach’s alpha of 0.89 and 0.88 as well as 0.94, respectively [55, 65, 66]. Further, the CD-RISC showed high test-retest reliability over a 12-month follow-up period [67–69]. Cronbach’s alpha was 0.86 in our patient sample and 0.76 in our relative sample.

#### Hospital-related factors

We assessed several hospital-related factors through items specifically designed for this study. Patients and relatives were asked whether the hospital’s psychosocial care team was involved, the burden of having no visitors or not being able to visit (VAS 0–10) and missing physical closeness of their relatives (VAS 0–10).

Patients were further asked whether there was contradictory information, i.e., information from one treating team member did not match information from other treating team members, they received by the medical team (VAS 0–10) and the perceived competence of the treating physician (VAS 0–10).

Relatives were asked whether they were in contact with the medical team, the satisfaction with the communication with the medical team (VAS 0–10), whether they received information regarding the patient’s prognosis, whether patient’s medical care was perceived as sufficient or inadequate, the comprehensibility of medical information (VAS 0–10) and whether they received recommendations regarding own care.

### Outcome variables

#### Psychological distress

All outcome variables were collected 30 days after hospital discharge. Psychological distress, i.e., symptoms of anxiety and/or depression experienced by patients and relatives, was measured by the Hospital Anxiety and Depression Scale [HADS; 70]. Cronbach’s alpha was ≥0.80 for both the anxiety and depression subscale in both the patient and relative sample. In line with previous research, we used a cut-off value of ≥8, indicating moderately severe symptoms, and operationalized presence of psychological distress as a score of ≥8 (range: 0 to 21) on either the depression or the anxiety subscale of the HADS [70, 71]. The questionnaire was specifically developed for patients with physical disease and intentionally excludes items associated with physical symptoms to avoid confounding with psychopathological symptoms [70]. Good reliability and validity were shown for the HADS, with a Cronbach’s alpha of 0.83 and 0.82 for the subscales anxiety and depression, respectively, and an optimal balance between sensitivity and specificity of approximately 0.80 when applying a cut-off score of ≥8 on both subscales [71].

#### Symptoms of post-traumatic stress disorder

Further, symptoms of post-traumatic stress disorder were assessed through a German translation of the Impact of Event Scale-revised [IES-r; 72–74] which had a Cronbach’s alpha of ≥0.90 in both our patient and relative sample. The IES-r is a 22-item questionnaire which assesses symptoms of emotional distress caused by traumatic events and is divided into three subscales, i.e., intrusion, avoidance and hyperarousal. It is also applicable in general population samples and has been shown to have high internal consistency with a Cronbach’s alpha of 0.96 and good diagnostic accuracy when applying a cut-off score of 1.5 [75].

### Statistical analyses

Descriptive statistics, i.e., frequencies as well as means and standard deviations were used to display characteristics of the patient and relative sample. We stratified the two samples based on the psychological distress whereas a score of ≥8 on the anxiety and/or depression scale of the HADS was determined as presence of psychological distress and a score of <8 on both scales as absence of psychological distress.

We conducted all analyses separately for each the patient and the relative sample. We evaluated associations between potential predictors and outcomes, separately in two steps, through univariate and multivariate analyses. To account for missing data in predictors used in the multivariate analyses, we imputed datasets using multiple imputations by chained equations. Imputations were calculated using multiple covariables within domains also including main outcomes to reduce bias as previously suggested [76], i.e. for patients: age, gender, children, duration of hospitalization, Charlson Comorbidity Score, NEWS score, ICU stay, pre-existing psychological diagnoses, worries due to COVID-19 media reports, worries about uncertain prognosis, burden of isolation measures, worries about health of relatives, helpfulness of social contacts, helpfulness of distraction, CD-RISC-10, PSS-10, involvement of psychosocial care team, burden of having no visitors, missing physical closeness, and psychological distress (HADS); for relatives: age, gender, cultural background, religion, civil status, children, current job situation, relationship with patient, EuroQol VAS, pre-existing psychological diagnoses, psychotropic drugs, CD-RISC-10, PSS-10, worries due to COVID-19 media reports, worries about infection, worries about uncertain prognosis, contact with medical team, burden of having no visitors, missing physical closeness, and psychological distress (HADS). Model performance of imputed data was also compared to those of crude values to check consistency. We found a similar pattern when doing a full set analysis (see S1 and S2 Tables in S1 File).

First, we calculated univariate logistic regression models separately for patients and relatives. We further investigated the associations between each variable and psychological burden by adjusting each of these analyses for age, gender and study center. In a next step, we calculated a separate multivariate logistic regression model for each domain, resulting in four models in each sample. Each of these models included predefined factors from the respective domain, i.e., a) age for the sociodemographic model, b) duration of hospitalization, use of anxiolytics during hospitalization, and ICU stay for the illness-related factors model, c) burden of isolation measures due to COVID-19 and coping through social contacts in the psychosocial model, and d) burden of having no visitors and missing physical closeness in the hospital-related factors model. In addition, we included all factors that were significantly associated with psychological distress in the previous, age-, gender- and study center-adjusted analyses for each domain. Third, to evaluate which factors might be independently associated with psychological distress, we analyzed an overall model containing all factors that were significantly associated with psychological distress within the four domain models. We calculated odds ratios (OR) and 95% confidence intervals (CI). A p-value of < .05 (two-tailed) was considered significant. Areas under the curve (AUC) were created to evaluate the potential prognostic value of the factors regarding psychological distress. All statistical analyses were conducted using Stata 15 (Stata Corp, College Station, Texas, USA).

## Results

### Characteristics of the study sample

**Fig 1:**
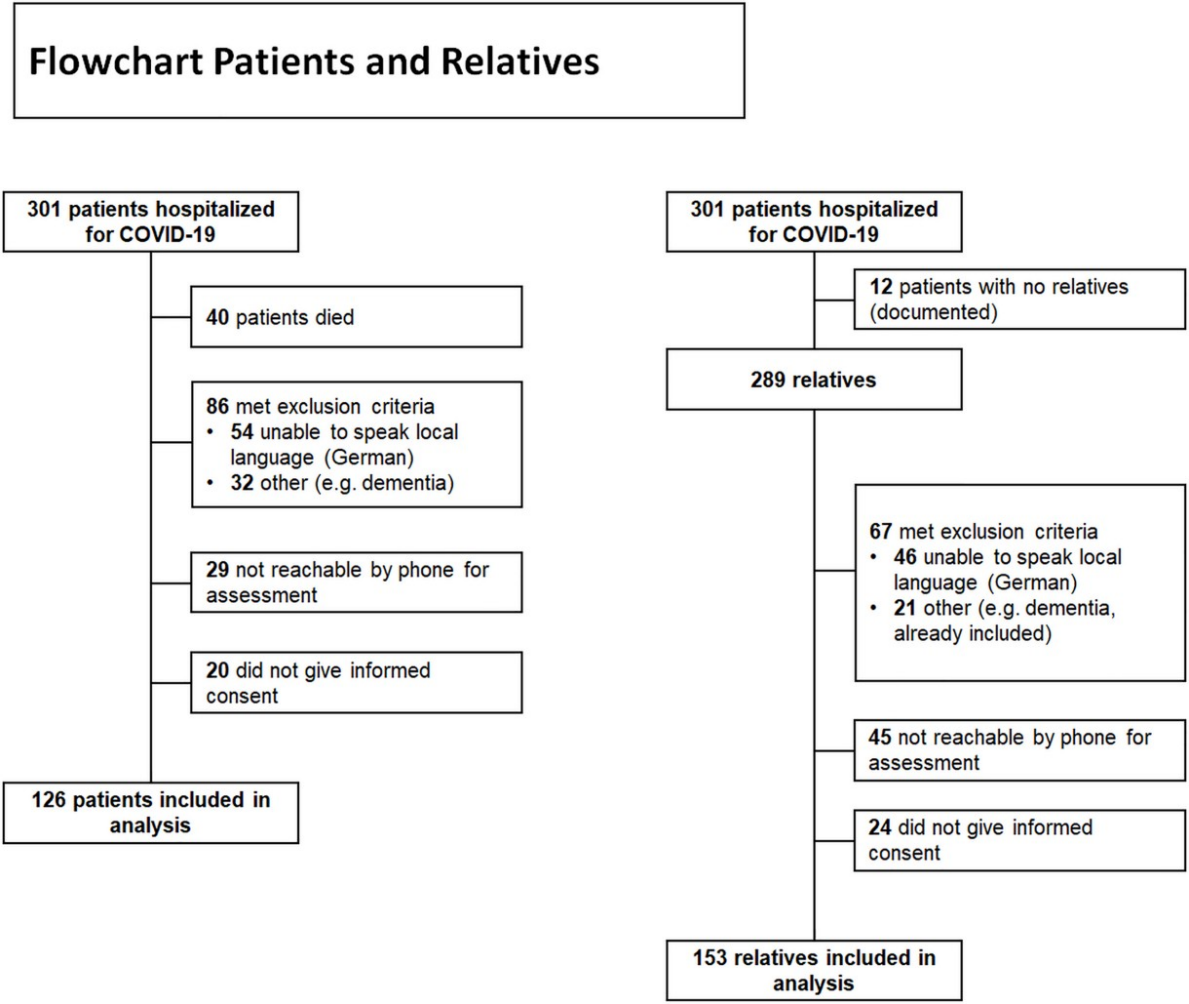
Flow diagram of the study population. Legend: Flow diagram illustrating inclusion and exclusion of eligible participants.

Between March and June 2020, a total of 301 patients with COVID-19 were hospitalized in the University Hospital Basel (n = 198) and the Kantonsspital Aarau (n = 103) (Fig 1). Forty of these patients (13.3%) died during hospitalization or within 30 days after discharge, 54 (17.9%) were unable to speak the local language (German), 32 (16.6%) met exclusion criteria such as dementia or severe underlying psychiatric conditions, 29 (9.6%) were not reachable by phone for assessment, and 20 (6.6%) did not give informed consent. In 12 (4%) of all 301 hospitalized patients no relatives were documented in the medical charts. As we identified and approached only one relative per patient, there were therefore 289 potentially eligible relatives left. Of these 289 relatives, 15.9% did not speak German and 7% were excluded due to other criteria, e.g., cognitive impairment or being already included in the patient sample. Forty-five (15.6%) were not reachable by phone and 24 (8.3%) did not give informed consent. Thus, the final cohort consisted of 126 patients and 153 relatives. Table 1 shows sociodemographic and clinical characteristics of the participants.

**Table 1:** Sociodemographic and clinical characteristics the study populations.

Sociodemographic and clinical characteristics		Patients	Relatives
n		126	153
Age (years)		58.2 (16.35)	57.7 (14.94)
Gender (female)		50 (39.7%)	115 (75.2%)
Citizenship	Switzerland	86 (68.3%)	125 (81.7%)
	Germany	14 (11.1%)	7 (4.6%)
	France	5 (4.0%)	6 (3.9%)
	Other	21 (16.7%)	16 (10.5%)
Cultural background	Central Europe	89 (70.6%)	113 (73.9%)
	Western Europe	11 (8.7%)	8 (5.2%)
	Southern Europe	16 (12.7%)	18 (11.8%)
	Northern Europe	2 (1.6%)	5 (3.3%)
	Asia	4 (3.2%)	4 (2.6%)
	Other	4 (3.2%)	5 (3.3%)
Religious affiliation	Catholic	33 (26.4%)	46 (30.3%)
	Protestant	32 (25.6%)	48 (31.6%)
	Other Christian denomination	9 (7.2%)	10 (6.6%)
	Jewish	2 (1.6%)	2 (1.3%)
	Muslim	11 (8.8%)	8 (5.3%)
	Other religion	3 (2.4%)	3 (2.0%)
	No religious affiliation	35 (28.0%)	35 (23.0%)
Civil status	Married/in partnership	80 (63.5%)	110 (72.4%)
	Divorced	22 (17.5%)	14 (9.2%)
	Widowed	9 (7.1%)	12 (7.9%)
	Single	15 (11.9%)	16 (10.5%)
Children, yes		86 (70.5%)	114 (74.5%)
Education	High School	13 (10.7%)	7 (4.6%)
	Apprenticeship	83 (68.6%)	99 (65.6%)
	College/University	25 (20.7%)	45 (29.8%)
Current job situation	Employed	72 (57.6%)	81 (53.3%)
	Unemployed	1 (0.8%)	4 (2.6%)
	Retired	42 (33.6%)	56 (36.8%)
	Disability benefits	6 (4.8%)	3 (2.0%)
	Homemaker	2 (1.6%)	7 (4.6%)
	Other	2 (1.6%)	1 (0.7%)
Previous psychological therapy		7 (5.7%)	7 (4.7%)
Pre-existing psychological comorbidities		18 (14.8%)	18 (12.1%)
**Patient characteristics**			
Duration of hospitalization (days), mean (SD)		9.00 (6.49)	
Severity of illness (NEWS score), mean (SD)		6.21 (3.71)	
Comorbidity (CCI), mean (SD)		2.40 (2.17)	
Antibiotics during hospitalization		39 (31.2%)	
Oxygen supply	No oxygen supply	49 (38.9%)	
	Nasal cannula/NIV	65 (51.6%)	
	Intubation	12 (9.5%)	
Anxiolytics during hospitalization		21 (16.9%)	
Investigational treatment^a^		85 (68.0%)	
ICU stay (yes/no)		19 (15.1%)	
**Relative characteristics**			
Relationship to patient	Patient is partner		77 (50.3%)
	Patient is child		12 (7.8%)
	Patient is sibling		15 (9.8%)
	Patient is parent		37 (24.2%)
	Other		12 (7.8%)
Relative living in same household with patient			83 (54.2%)
Patient died (bereaved relatives)			26 (17%)
Relative quarantined			64 (48.5%)
Relative also infected with COVID-19			51 (34.5%)

Data are presented as n (%) or mean (standard deviation).

Abbreviations: SD, standard deviation; NEWS, National Early Warning Score; NIV, Non-invasive ventilation; ICU, Intensive Care Unit; CCI, Charlson Comorbidity Index

^a^ Investigational treatment: Hydroxychloroquine, Lopinavir/Ritonavir, Remdesivir, Tocilizumab, Convalescent Plasma

### Psychological distress in patients 30 days after discharge

Twenty-four patients (19.1%) showed psychological distress, i.e., symptoms of depression and/or anxiety. Of those, 22 (17.5%) patients showed symptoms of anxiety and 10 (7.9%) showed symptoms of depression.

Table 2A and 2B give a detailed overview of the associations with psychological distress for patients.

**Table 2:** Factors associated with psychological distress in patients.

a.							
		**No psychological distress**	**Psychological distress**	**Univariate OR (95%CI)**	***p***	**Age, gender, center adjusted OR (95%CI)**	***p***
		n = 102	n = 24				
**Sociodemographic factors**							
Age (years)		58.62 (16.10)	56.63 (17.65)	0.99 (0.97, 1.02)	0.590		
Gender	male	68 (66.7%)	8 (33.3%)	1 (Ref)		1 (Ref)	
	female	34 (33.3%)	16 (66.7%)	4 (1.56, 10.27)	**0.004**		
Citizenship	Swiss	71 (69.6%)	15 (62.5%)	1 (Ref)		1 (Ref)	
	Non-Swiss	31 (30.4%)	9 (37.5%)	1.37 (0.54, 3.48)	0.502	1.47 (0.52, 4.11)	0.464
Cultural background	Central/Western Europe	84 (82.4%)	16 (66.7%)	1 (Ref)		1 (Ref)	
	Other	18 (17.6%)	8 (33.3%)	2.33 (0.87, 6.28)	0.093	3.55 (1.03, 12.27)	**0.045**
Religious affiliation	Christian	61 (59.8%)	13 (56.5%)	1 (Ref)		1 (Ref)	
	Non-Christian religion	9 (8.8%)	7 (30.4%)	3.65 (1.15, 11.58)	**0.028**	5.51 (1.38, 22.06)	**0.016**
	No religious affiliation	32 (31.4%)	3 (13.0%)	0.44 (0.12, 1.66)	0.225	0.36 (0.09, 1.43)	0.146
Civil status	Married/ Partnership	66 (64.7%)	14 (58.3%)	1 (Ref)		1 (Ref)	
	Widowed/ separated/single	36 (35.3%)	10 (41.7%)	1.31 (0.53, 3.24)	0.560	0.68 (0.23, 1.94)	0.468
Children	no	30 (30%)	6 (26%)	1 (Ref)		1 (Ref)	
	yes	69 (70%)	17 (74%)	1.23 (0.44, 3.43)	0.690	1.54 (0.51, 4.68)	0.449
Current job situation	Employed	40 (39.6%)	13 (54.2%)	1 (Ref)		1 (Ref)	
	Not employed	43 (45%)	12 (50%)	1.8 (0.74, 4.42)	0.198	2.9 (0.86, 9.74)	0.085
**Illness-related factors**							
Time point of COVID-19 diagnosis^a^, mean (SD)		29.98 (12.39)	33.38 (7.56)	1.03 (0.99, 1.06)	0.202	1.06 (1.01, 1.11)	**0.016**
Duration of hospitalization (days), mean (SD)		9.45 (6.86)	7.08 (4.16)	0.93 (0.84, 1.02)	0.117	0.94 (0.86, 1.04)	0.247
Severity of illness (NEWS score), mean (SD)		6.25 (3.71)	6.04 (3.77)	0.99 (0.87, 1.11)	0.813	1.08 (0.92, 1.26)	0.367
Comorbidity (CCI), mean (SD)		2.44 (2.18)	2.25 (2.17)	0.96 (0.78, 1.18)	0.697	1.01 (0.69, 1.47)	0.964
Self-perceived overall health status (Euroqol), mean (SD)		75.98 (16.30)	65.25 (19.91)	0.97 (0.94, 0.99)	**0.009**	0.97 (0.94, 1)	**0.023**
Antibiotics during hospitalization	no	70 (69.3%)	16 (66.7%)	1 (Ref)		1 (Ref)	
	yes	31 (30.7%)	8 (33.3%)	1.13 (0.44, 2.91)	0.802	1.41 (0.51, 3.92)	0.510
Investigational therapy	no	32 (31.7%)	8 (33.3%)	1 (Ref)		1 (Ref)	
	yes	69 (68.3%)	16 (66.7%)	0.93 (0.36, 2.39)	0.876	1.14 (0.38, 3.38)	0.812
Anxiolytics during hospitalization	no	86 (85.1%)	17 (73.9%)	1 (Ref)		1 (Ref)	
	yes	15 (14.9%)	6 (26.1%)	2.02 (0.69, 5.96)	0.201	2.06 (0.65, 6.52)	0.220
ICU stay	no	85 (83.3%)	22 (91.7%)	1 (Ref)		1 (Ref)	
	yes	17 (16.7%)	2 (8.3%)	0.45 (0.1, 2.12)	0.315	0.61 (0.12, 3.01)	0.542
Intubation	no	91 (89.2%)	23 (95.8%)	1 (Ref)		1 (Ref)	
	yes	11 (10.8%)	1 (4.2%)	0.36 (0.04, 2.93)	0.339	0.49 (0.06, 4.3)	0.518
**Psychosocial factors**							
Pre-existing psychological comorbidities	no	90 (92%)	14 (58%)	1 (Ref)		1 (Ref)	
	yes	8 (8%)	10 (42%)	8.04 (2.71, 23.83)	**<0.001**	5.73 (1.77, 18.59)	**0.004**
Psychotropic drugs	no	90 (92%)	19 (79%)	1 (Ref)		1 (Ref)	
	yes	8 (8%)	5 (21%)	2.96 (0.87, 10.05)	0.082	2.51 (0.67, 9.4)	0.171
Resilience (CD-RISC), mean (SD)		32.79 (4.69)	24.53 (7.94)	0.79 (0.71, 0.89)	**<0.001**	0.8 (0.71, 0.91)	**<0.001**
Perceived Stress (PSS), mean (SD)		20.95 (6.43)	29.64 (9.64)	1.17 (1.06, 1.3)	**0.002**	1.18 (1.06, 1.32)	**0.003**
Self-perceived stigmatization (VAS 0–10), mean (SD)		2.49 (3.10)	3.15 (3.53)	1.06 (0.92, 1.24)	0.408	1.05 (0.9, 1.23)	0.507
Consumption of COVID-19 media reports	no	15 (15%)	6 (25%)	1 (Ref)		1 (Ref)	
	yes	82 (85%)	18 (75%)	0.55 (0.19, 1.61)	0.274	0.71 (0.22, 2.28)	0.561
Duration of COVID-19 media consumption, mean (SD)		34.76 (30.51)	40.79 (30.24)	1.01 (0.99, 1.02)	0.436	1.01 (0.99, 1.03)	0.239
Worries due to COVID-19 media reports, mean (SD)		3.56 (2.84)	6.00 (3.79)	1.28 (1.09, 1.5)	**0.002**	1.23 (1.05, 1.45)	**0.012**
Frequency of contacts with relatives	Daily	88 (89%)	16 (67%)	1 (Ref)			
	Less than daily	11 (11%)	8 (33%)	4 (1.39, 11.49)	**0.010**	5.13 (1.6, 16.47)	**0.006**
Type of communication between patients and relatives	Telephone, text and other	58 (59%)	14 (58%)	1 (Ref)			
	Video calls and visits	41 (41%)	10 (42%)	1.01 (0.41, 2.5)	0.982	0.91 (0.29, 2.81)	0.869
*Current worries and burdens (VAS 0–10)*							
Worried about uncertain prognosis, mean (SD)		5.23 (3.16)	7.04 (3.11)	1.22 (1.04, 1.45)	**0.017**	1.22 (1.03, 1.45)	**0.022**
Burden of isolation measures, mean (SD)		4.67 (3.63)	6.63 (3.32)	1.17 (1.02, 1.34)	**0.022**	1.15 (0.99, 1.33)	0.074
Burden of boredom, mean (SD)		2.96 (3.41)	3.52 (3.36)	1.05 (0.92, 1.2)	0.474	1.03 (0.9, 1.18)	0.683
Worried about health of relatives, mean (SD)		4.36 (3.59)	7.30 (3.10)	1.3 (1.11, 1.52)	**0.001**	1.32 (1.1, 1.57)	**0.002**
Burden of missing relatives, mean (SD)		5.15 (3.61)	6.00 (3.94)	1.07 (0.94, 1.22)	0.326	1.03 (0.89, 1.19)	0.695
Worried about job situation, mean (SD)		1.29 (2.66)	1.91 (3.74)	1.07 (0.93, 1.24)	0.360	1.03 (0.88, 1.22)	0.694
Worried about finances, mean (SD)		0.88 (2.22)	1.78 (3.34)	1.13 (0.96, 1.33)	0.129	1.1 (0.93, 1.31)	0.255
Worried about medical care, mean (SD)		0.55 (1.52)	0.57 (1.50)	1.01 (0.74, 1.36)	0.973	0.95 (0.69, 1.31)	0.761
Other worries, mean (SD)		1.71 (3.42)	1.70 (3.57)	1 (0.87, 1.15)	0.993	1.01 (0.87, 1.18)	0.894
*Helpfulness of coping strategies (VAS 0–10)*							
Social contacts, mean (SD)		7.79 (2.65)	6.82 (2.99)	0.89 (0.76, 1.04)	0.139	0.85 (0.72, 1.02)	0.074
Distraction, mean (SD)		5.72 (3.52)	4.38 (3.59)	0.9 (0.77, 1.05)	0.174	0.89 (0.76, 1.04)	0.148
Tranquilizers, mean (SD)		0.46 (1.90)	1.36 (3.23)	1.16 (0.91, 1.48)	0.242	1.15 (0.88, 1.51)	0.301
Other, mean (SD)		5.45 (4.31)	6.23 (4.40)	1.04 (0.9, 1.21)	0.552	1.04 (0.89, 1.21)	0.634
**Hospital-related factors (VAS 0–10)**							
Involvement of psychosocial care team	no	90 (90%	18 (75%)	1 (Ref)		1 (Ref)	
	yes	10 (10.0%)	6 (25.0%)	3 (0.97, 9.3)	0.057	3.1 (0.88, 10.89)	0.078
Contradictory information given by medical team, mean (SD)		0.94 (2.02)	1.17 (1.85)	1.06 (0.85, 1.31)	0.620	0.97 (0.76, 1.23)	0.794
Perceived competence of treating physician, mean (SD)		8.77 (1.41)	8.48 (2.41)	0.91 (0.7, 1.17)	0.460	0.94 (0.71, 1.25)	0.666
Burden of having no visitors, mean (SD)		3.46 (3.34)	5.08 (3.88)	1.14 (1, 1.3)	**0.044**	1.1 (0.96, 1.26)	0.162
Missing physical closeness, mean (SD)		4.33 (3.73)	5.96 (3.77)	1.13 (0.99, 1.27)	0.062	1.1 (0.96, 1.25)	0.160
**b.**							
		**Multivariate model within domains**		**Overall multivariate model**			
		**OR (95%CI)**	***p***	**OR (95%CI)**	***p***		
**Sociodemographic factors**							
Age (years)		0.98 (0.95, 1.02)	0.332				
Gender	male	1 (Ref)		1 (Ref)			
	female	5.6 (1.9, 16.5)	**0.002**	1.7 (0.38, 7.71)	0.49		
Cultural background	Central/Western Europe	1 (Ref)					
	Other	1.08 (0.21, 5.54)	0.926				
Religious affiliation	Christian	1 (Ref)		1 (Ref)			
	Non-Christian religion	6.06 (1.1, 33.29)	**0.038**	3.6 (0.38, 33.67)	0.262		
	No religious affiliation	0.35 (0.08, 1.44)	0.144	1.24 (0.19, 7.94)	0.82		
Current job situation	Employed	1 (Ref)		1 (Ref)			
	Not employed	3.84 (1.03, 14.25)	**0.044**	2.99 (0.63, 14.16)	0.169		
**Illness-related factors**							
Time point of COVID-19 diagnosis^a^, mean (SD)		1.03 (0.98, 1.07)	0.246				
Duration of hospitalization (days), mean (SD)		0.9 (0.8, 1.01)	0.084				
Self-perceived overall health status (Euroqol), mean (SD)		0.96 (0.94, 0.99)	**0.008**	0.98 (0.94, 1.02)	0.383		
Anxiolytics during hospitalization	no	1 (Ref)					
	yes	2.71 (0.79, 9.31)	0.112				
ICU stay	no	1 (Ref)					
	yes	1 (0.16, 6.12)	0.996				
**Psychosocial factors**							
Pre-existing psychological comorbidities	no	1 (Ref)					
	yes	5.41 (0.85, 34.35)	0.073				
Resilience (CD-RISC), mean (SD)		0.83 (0.71, 0.97)	**0.017**	0.82 (0.71, 0.94)	**0.005**		
Perceived Stress (PSS), mean (SD)		1.23 (1.06, 1.42)	0.006	1.21 (1.06, 1.38)	**0.006**		
Worries due to COVID-19 media reports, mean (SD)		1.31 (0.99, 1.72)	0.057				
Frequency of contacts with relatives	Daily	1 (Ref)		1 (Ref)			
	Less than daily	9.57 (1.8, 50.91)	**0.008**	7.67 (1.42, 41.58)	**0.018**		
*Current worries and burdens (VAS 0–10)*							
Burden of isolation measures, mean (SD)		0.95 (0.72, 1.24)	0.680				
Worried about health of relatives, mean (SD)		1.12 (0.9, 1.4)	0.312				
*Helpfulness of coping strategies (VAS 0–10)*							
Social contacts, mean (SD)		0.74 (0.56, 0.99)	**0.04**	0.76 (0.57, 1.01)	0.056		
**Hospital-related factors (VAS 0–10)**							
Involvement of psychosocial care team	no	1 (Ref)					
	yes	2.59 (0.8, 8.31)	0.111				
Burden of having no visitors, mean (SD)		1.06 (0.86, 1.3)	0.575				
Missing physical closeness, mean (SD)		1.07 (0.88, 1.3)	0.52				

Data are presented as n (%) or mean (standard deviation)

^a^consecutive days, starting with day 0 for first patients hospitalized

Abbreviations: SD, standard deviation; OR, odds ratio; 95%CI, 95% Confidence Interval; COVID-19, Coronavirus disease 2019; CD-RISC, Connor-Davidson Resilience Scale; PSS, Perceived Stress Scale; VAS, visual analogue scale

#### Factors associated with psychological distress in patients

Several factors were associated with psychological distress in univariate models, including sociodemographic factors, i.e., patient gender, religious affiliation, illness-related factors, i.e., self-perceived overall health status, psychosocial factors, i.e., pre-existing psychological comorbidities, resilience, perceived stress, worries due to COVID-19 media reports, frequency of contact with relatives, worries about uncertain prognosis, burden of isolation measures due to COVID-19, worries about health of relatives, and hospital-related factors, i.e., burden of having no visitors. All these variables except from burden of isolation measures and having no visitors were still significantly associated when these analyses were each adjusted for age, gender and study center. Additionally, cultural background and time point of COVID-19 diagnosis were significantly associated with psychological distress.

In a next step, we evaluated all variables significantly associated in these adjusted analyses as well as several predefined variables within four domain models. The results are presented in Table 2B.

The sociodemographic domain model with an area under the receiver-operating characteristic curve (AUC) of 0.77, included the variables age, gender, cultural background, religious affiliation, and current job situation. Of these, being female, non-Christian religion and no employment were independently associated with increased likelihood of psychological distress. In the illness-related factors model containing timepoint of COVID-19 diagnosis, duration of hospitalization, self-perceived overall health status, anxiolytics during hospitalization, and ICU stay (AUC of 0.72), only lower self-perceived overall health status was independently associated. Of the variables pre-existing psychological comorbidities, resilience, perceived stress, worries due to COVID-19 media reports, frequency of contacts with relatives, burden of isolation measures, worries about health of relatives, and social contacts as a coping strategy in the psychosocial domain model (AUC of 0.95), lower resilience, higher perceived stress, lower frequency of contacts with relatives, and lower perceived helpfulness of social contacts as a coping strategy, were each independently associated with higher likelihood of psychological distress. None of the variables, involvement of psychosocial care team, burden of having no visitors and missing physical closeness were independently associated in the fourth domain model (AUC of 0.67).

After including all factors independently associated within these four domain models in a final overall model, only resilience, perceived stress and less than daily frequency of contact with relatives remained independently associated with psychological distress. A model including these three independently associated variables showed very good discrimination regarding presence or absence of psychological distress in patients hospitalized with COVID-19, with an AUC of 0.92.

### Psychological distress in relatives 30 days after discharge

In the relative sample, 35 participants (22.9%) met the criteria for psychological distress, i.e., showed symptoms of depression and/or anxiety defined by a score of ≥8 on the depression and/or anxiety subscale of the HADS. Of those, 25 had symptoms of anxiety (16.3%) and 23 had symptoms of depression (15%).

Table 3A and 3B provide an overview of the different variables and associations with psychological distress.

**Table 3:** Factors associated with psychological distress in relatives.

a.							
		**No Psychological distress**	**Psychological distress**	**Univariate model, OR (95%CI)**	***p***	**Age, gender, center adjusted model, OR (95%CI)**	***p***
		n = 118	n = 35				
**Sociodemographic factors**							
Age (years)		56.98 (14.91)	60.09 (15.01)	1.01 (0.99, 1.04)	0.281		
Gender	male	31 (26.3%)	7 (20.0%)	1 (Ref)			
	female	87 (73.7%)	28 (80.0%)	1.43 (0.57, 3.59)	0.452		
Citizenship	Swiss	96 (81.4%)	29 (82.9%)	1 (Ref)		1 (Ref)	
	Non-Swiss	22 (18.6%)	6 (17.1%)	0.9 (0.33, 2.44)	0.840	0.98 (0.35, 2.75)	0.966
Cultural background	Central/Western Europe	93 (78.8%)	28 (80.0%)	1 (Ref)		1 (Ref)	
	Other	25 (21.2%)	7 (20.0%)	0.93 (0.36, 2.38)	0.880	1.11 (0.39, 3.18)	0.842
Religious affiliation	Christian	79 (67.5%)	25 (71.4%)	1 (Ref)		1 (Ref)	
	Non-Christian religion	10 (8.5%)	3 (8.6%)	0.95 (0.24, 3.72)	0.939	1.03 (0.24, 4.42)	0.963
	No religious affiliation	28 (23.9%)	7 (20.0%)	0.79 (0.31, 2.03)	0.624	0.83 (0.31, 2.19)	0.705
Civil status	Married/ Partnership	87 (74.4%)	23 (65.7%)	1 (Ref)		1 (Ref)	
	Widowed/ separated/single	30 (25.6%)	12 (34.3%)	1.51 (0.67, 3.41)	0.318	1.5 (0.66, 3.41)	0.332
Children	no	35 (29.7%)	4 (11.4%)	1 (Ref)		1 (Ref)	
	yes	83 (70.3%)	31 (88.6%)	3.27 (1.07, 9.95)	**0.037**	3.16 (1.02, 9.81)	**0.046**
Current job situation	Employed	69 (58.5%)	12 (35.3%)	1 (Ref)		1 (Ref)	
	Not employed	49 (41.5%)	22 (64.7%)	2.58 (1.17, 5.71)	**0.019**	2.97 (1.07, 8.3)	**0.037**
**Illness-related factors**							
Relative quarantined	no	55 (56%)	13 (39%)	1 (Ref)		1 (Ref)	
	yes	44 (44%)	20 (61%)	1.92 (0.86, 4.29)	0.110	1.94 (0.86, 4.37)	0.110
Relative ill with COVID-19	no	73 (63.5%)	24 (72.7%)	1 (Ref)		1 (Ref)	
	yes	42 (36.5%)	9 (27.3%)	0.65 (0.28, 1.53)	0.326	0.72 (0.3, 1.71)	0.455
Self-perceived overall health status (Euroqol), mean (SD)		84.89 (13.28)	70.41 (20.73)	0.95 (0.93, 0.97)	**<0.001**	0.95 (0.93, 0.97)	**<0.001**
Time point of COVID-19 diagnosis^a^, mean (SD)		30.04 (12.06)	30.26 (13.99)	1 (0.97, 1.03)	0.929	1 (0.97, 1.04)	0.774
Death of patient	no	103 (87.3%)	24 (68.6%)	1 (Ref)		1 (Ref)	
	yes	15 (12.7%)	11 (31.4%)	3.15 (1.28, 7.71)	**0.012**	3.8 (1.37, 10.55)	**0.010**
**Psychosocial factors**							
Relationship with patient	Patient is partner	60 (50.8%)	17 (48.6%)	1 (Ref)		1 (Ref)	
	Patient is child	6 (5.1%)	6 (17.1%)	3.53 (1.01, 12.36)	**0.049**	3.16 (0.88, 11.39)	0.079
	Patient is parent	28 (23.7%)	9 (25.7%)	1.13 (0.45, 2.86)	0.789	1.55 (0.54, 4.47)	0.417
	Other	24 (20.3%)	3 (8.6%)	0.44 (0.12, 1.64)	0.223	0.42 (0.11, 1.59)	0.203
Relative living in same household with patient	no	54 (45.8%)	16 (45.7%)	1 (Ref)		1 (Ref)	
	yes	64 (54.2%)	19 (54.3%)	1 (0.47, 2.14)	0.996	0.97 (0.45, 2.08)	0.929
Frequency of contact with patient	Daily	74 (63.2%)	23 (65.7%)	1 (Ref)		1 (Ref)	
	Less than daily	43 (36.8%)	12 (34.3%)	0.9 (0.41, 1.98)	0.790	0.95 (0.42, 2.11)	0.892
Relative sought out psychological help	no	109 (95.6%)	33 (94.3%)	1 (Ref)		1 (Ref)	
	yes	5 (4.4%)	2 (5.7%)	1.32 (0.24, 7.13)	0.746	1.29 (0.23, 7.11)	0.774
Pre-existing psychological comorbidities	no	101 (88.6%)	30 (85.7%)	1 (Ref)		1 (Ref)	
	yes	13 (11.4%)	5 (14.3%)	1.29 (0.43, 3.93)	0.648	1.18 (0.38, 3.66)	0.772
Psychotropic drugs	no	104 (92.0%)	26 (74.3%)	1 (Ref)		1 (Ref)	
	yes	9 (8.0%)	9 (25.7%)	4 (1.44, 11.08)	**0.008**	3.83 (1.35, 10.9)	**0.012**
Resilience (CD-RISC), mean (SD)		31.93 (4.32)	27.56 (7.18)	0.86 (0.79, 0.94)	**<0.001**	0.86 (0.78, 0.94)	**0.001**
Perceived Stress (PSS), mean (SD)		21.95 (5.87)	28.30 (9.07)	1.14 (1.06, 1.23)	**<0.001**	1.18 (1.08, 1.28)	**<0.001**
Type of communication between relatives and patients	Telephone, text and other	77 (69.4%)	13 (40.6%)	1 (Ref)		1 (Ref)	
	Video calls & visits	34 (30.6%)	19 (59.4%)	3.31 (1.47, 7.46)	**0.004**	3.68 (1.58, 8.58)	**0.002**
Consumption of COVID-19 media reports	no	6 (8%)	2 (8%)	1 (Ref)		1 (Ref)	
	yes	74 (93%)	22 (92%)	0.89 (0.17, 4.74)	0.893	0.7 (0.12, 4.09)	0.696
Duration of COVID-19 media consumption, mean (SD)		54.44 (48.96)	60.24 (67.02)	1 (0.99, 1.01)	0.669	1 (0.99, 1.01)	0.868
Worries due to COVID-19 media reports, mean (SD)		5.00 (3.12)	6.22 (3.04)	1.14 (0.97, 1.34)	0.105	1.13 (0.97, 1.33)	0.118
*Current worries and burdens (VAS 0–10)*							
Perceived overall burden due to COVID-19, mean (SD)		5.16 (2.91)	8.24 (2.05)	1.66 (1.33, 2.06)	**<0.001**	1.76 (1.39, 2.23)	**<0.001**
Worried about uncertain prognosis, mean (SD)		4.87 (3.38)	6.59 (3.91)	1.16 (1.02, 1.32)	**0.024**	1.19 (1.03, 1.37)	**0.015**
Worried about infection, mean (SD)		2.59 (2.83)	4.09 (3.95)	1.15 (1.02, 1.3)	**0.021**	1.18 (1.04, 1.34)	**0.013**
Burden of isolation measures, mean (SD)		3.94 (3.12)	7.19 (3.31)	1.38 (1.19, 1.6)	**<0.001**	1.39 (1.19, 1.62)	**<0.001**
Burden of separation from patient, mean (SD)		5.62 (3.21)	7.29 (3.49)	1.19 (1.03, 1.36)	**0.017**	1.23 (1.06, 1.42)	**0.008**
Other worries, mean (SD)		6.17 (4.27)	7.95 (3.46)	1.13 (0.98, 1.3)	0.096	1.12 (0.97, 1.3)	0.120
*Helpfulness of coping strategies (VAS 0–10)*							
Social contacts, mean (SD)		7.80 (2.74)	7.94 (2.33)	1.02 (0.88, 1.19)	0.799	1.04 (0.88, 1.24)	0.626
Distraction, mean (SD)		6.37 (3.54)	6.68 (3.11)	1.03 (0.91, 1.16)	0.657	1.03 (0.91, 1.18)	0.609
Tranquilizers, mean (SD)		0.78 (2.25)	1.67 (3.06)	1.13 (0.98, 1.31)	0.094	1.13 (0.97, 1.31)	0.111
Alcohol consumption, mean (SD)		0.66 (1.70)	0.07 (0.37)	0.5 (0.2, 1.24)	0.133	0.5 (0.2, 1.25)	0.137
Relaxation techniques, mean (SD)		2.62 (3.78)	2.58 (3.69)	1 (0.9, 1.11)	0.961	1.01 (0.89, 1.13)	0.933
Sports, mean (SD)		5.23 (4.16)	2.43 (3.65)	0.84 (0.75, 0.94)	**0.002**	0.82 (0.72, 0.93)	**0.002**
Other, mean (SD)		7.94 (3.64)	6.60 (4.10)	0.92 (0.81, 1.04)	0.166	0.91 (0.8, 1.03)	0.130
**Hospital-related factors**							
Involvement of psychosocial care team	no	108 (95.6%)	30 (85.7%)	1 (Ref)		1 (Ref)	
	yes	5 (4.4%)	5 (14.3%)	3.6 (0.98, 13.26)	0.054	3.22 (0.83, 12.5)	0.091
Relative was in contact with medical team	no	49 (43.8%)	8 (22.9%)	1 (Ref)		1 (Ref)	
	yes	63 (56.3%)	27 (77.1%)	2.62 (1.1, 6.28)	**0.030**	2.86 (1.18, 6.93)	**0.020**
Satisfaction with communication with medical team, mean (SD)		7.98 (2.83)	8.36 (2.58)	1.05 (0.88, 1.26)	0.562	1.01 (0.83, 1.23)	0.915
Relative received information regarding prognosis	no	34 (53%)	7 (27%)	1 (Ref)		1 (Ref)	
	yes	30 (47%)	19 (73%)	3.08 (1.14, 8.33)	**0.027**	3.79 (1.33, 10.79)	**0.012**
Medical care was perceived as	Sufficient	52 (81%)	19 (76%)	1 (Ref)		1 (Ref)	
	Inadequate	12 (19%)	6 (24%)	1.37 (0.45, 4.16)	0.580	1.55 (0.48, 5.05)	0.468
Comprehensibility of medical information, mean (SD)		8.22 (2.99)	8.54 (2.50)	1.04 (0.88, 1.24)	0.636	0.99 (0.81, 1.21)	0.909
Relative received recommendations regarding own care	no	43 (67%)	17 (68%)	1 (Ref)		1 (Ref)	
	yes	21 (33%)	8 (32%)	0.96 (0.36, 2.59)	0.941	1.15 (0.41, 3.24)	0.785
Burden of not being able to visit patient (VAS 0–10), mean (SD)		5.78 (3.45)	7.65 (3.17)	1.2 (1.04, 1.37)	**0.010**	1.19 (1.03, 1.36)	**0.014**
Missing physical closeness (VAS 0–10), mean (SD)		4.92 (3.82)	7.06 (3.79)	1.17 (1.04, 1.31)	**0.009**	1.16 (1.03, 1.31)	**0.015**
**b.**							
		**Multivariate model within domains**			**Overall multivariate model**		
		**OR (95% CI)**		***p***	**OR (95% CI)**		* ****p***
**Sociodemographic factors**							
Children	no	1 (Ref)			1 (Ref)		
	yes	3.37 (1.09, 10.4)		**0.035**	2.91 (0.72, 11.73)		0.132
Current job situation	Employed	1 (Ref)			1 (Ref)		
	Not employed	2.45 (1.11, 5.39)		**0.027**	1.47 (0.51, 4.21)		0.473
**Illness-related factors**							
Relative quarantined	no	1 (Ref)					
	yes	1.98 (0.85, 4.61)		0.111			
Self-perceived overall health status (Euroqol), mean (SD)		0.95 (0.93, 0.98)		**<0.001**	0.97 (0.94, 1.01)		0.131
Death of patient	no	1 (Ref)			1 (Ref)		
	yes	2.84 (1.06, 7.63)		**0.038**	1.14 (0.29, 4.45)		0.846
**Psychosocial factors**							
Relationship with patient	Patient is partner	1 (Ref)					
	Patient is child	1.92 (0.18, 20.87)		0.593			
	Patient is parent	1.89 (0.52, 6.92)		0.334			
	Other	0.33 (0.05, 2.01)		0.230			
Psychotropic drugs	no	1 (Ref)					
	yes	1.1 (0.21, 5.75)		0.913			
Resilience (CD-RISC), mean (SD)		0.81 (0.7, 0.94)		**0.005**	0.85 (0.75, 0.96)		**0.007**
Perceived Stress (PSS), mean (SD)		0.9 (0.79, 1.04)		0.145			
Type of communication between relatives and patients	Telephone, text and other	1 (Ref)					
	Video calls & visits	2.91 (0.89, 9.51)		0.078			
					
*Current worries and burdens (VAS 0–10)*							
Perceived overall burden due to COVID-19, mean (SD)		1.84 (1.36, 2.48)		**<0.001**	1.72 (1.31, 2.25)		**<0.001**
Worried about uncertain prognosis, mean (SD)		1 (0.81, 1.22)		0.964			
Worried about infection, mean (SD)		1.19 (0.98, 1.46)		0.079			
Burden of isolation measures, mean (SD)		1.22 (0.97, 1.53)		0.087			
Burden of separation from patient, mean (SD)		0.89 (0.69, 1.16)		0.386			
*Helpfulness of coping strategies (VAS 0–10)*							
Sports, mean (SD)		0.81 (0.68, 0.97)		**0.018**	0.89 (0.78, 1.02)		0.100
**Hospital-related factors**							
Relative was in contact with medical team	no	1 (Ref)					
	yes	2.46 (0.97, 6.22)		0.057			
Relative received information regarding prognosis	no	1 (Ref)					
	yes	2.3 (0.99, 5.34)		0.053			
Burden of not being able to visit patient (VAS 0–10), mean (SD)		1.17 (0.99, 1.39)		0.068			
Missing physical closeness (VAS 0–10), mean (SD)		1.1 (0.95, 1.27)		0.208			

Data presented as n (%) or mean (standard deviation)

^a^consecutive days, starting with day 0 for first patients hospitalized

Abbreviations: SD, standard deviation; OR, Odds Ratio; 95% CI, 95% Confidence Interval; COVID-19, Coronavirus disease 2019; CD-RISC, Connor-Davidson Resilience Scale; PSS, Perceived Stress Scale

In univariate models (Table 3A), we found several factors associated with psychological distress, including sociodemographic factors, i.e., having children, not being employed, illness-related factors, i.e., lower self-perceived overall health status, death of patient, psychosocial factors, i.e., use of psychotropic drugs, lower resilience, higher perceived stress, communicating through video calls or being able to visit the patient, higher perceived overall burden, increased worries about uncertain diagnosis and infection, higher burden of isolation measures and separation from patient, sport as coping strategy, and hospital-related factors, i.e., relative was in contact with medical team, received information regarding prognosis, higher burden of not being able to visit patient, and missing physical closeness. Each of these factors remained significantly associated with psychosocial distress when adjusted for age, gender and study center.

In the multivariate analyses, several independently related factors emerged as illustrated in Table 3B. In the sociodemographic domain model, each of the two included variables, i.e., having children and not being employed, was associated with psychological distress. The AUC of this model was 0.66. In the illness-related factors model including self-perceived stress, if the relative was in quarantine and if the patient had died (AUC of 0.77), lower perceived overall health status and death of the patient were independently associated. In the psychosocial model, i.e., relationship with patient, psychotropic drugs, resilience, perceived stress, type of communication, perceived overall burden, worries about uncertain prognosis and infection, burden of isolation measures and separation from patient as well as sport as coping strategy, with an AUC of 0.92, higher resilience, higher perceived overall burden and helpfulness of sport as coping strategy were associated with psychological distress above and beyond the effects of the other factors in the model. The hospital-related factors model included the variables contact with medical team, receiving information regarding prognosis, burden of not being able to visit the patient and missing physical closeness (AUC 0.77). None of these were independently associated with the outcome.

In the final overall model containing all variables independently associated within the latter four domain models, only higher resilience and higher perceived overall burden caused by COVID-19 remained significantly, independently associated with the psychological distress. The model showed very good discrimination regarding relatives with and without psychological distress, with an AUC of 0.87.

### PTSD in patients and relatives 30 days after discharge

In total, 115 patients completed the IES-r questionnaire and could be included in the analyses. Ten patients (8.7%) showed considerable symptoms of PTSD. In univariate analyses, several factors in the domains of sociodemographic, psychosocial and hospital-related factors were associated with presence of clinically relevant symptoms of PTSD. In the sociodemographic domain these were lower age, female gender, non-swiss citizenship, non-central/western European cultural background, and non-Christian religion. In the psychosocial domain, lower resilience, higher perceived stress, increased worries due to COVID-19 media reports and about uncertain prognosis, as well as higher burden of isolation measures and of missing relatives each were associated with clinically relevant symptoms of PTSD. The hospital-related factors contradictory information given by medical team and higher burden of having no visitors were also associated. When age, gender and study center were added as covariates to each of these univariate analyses, only non-Swiss citizenship, non-central/western European background and higher worries due to COVID-19 media reports remained significant. A multivariate model containing these factors showed good discrimination, with an AUC of 0.84 (Table 4). The factor worries due to COVID-19 media reports was independently associated with clinically relevant symptoms of PTSD.

**Table 4:** Factors associated with high PTSD symptom levels in patients.

**a.**							
		**No/few PTSD symptoms**	**High PTSD symptom levels**	**Univariate model OR (95%CI)**	***p***	**Age, gender, center adjusted model, OR (95%CI)**	***p***
		n = 105	n = 10				
**Sociodemographic factors**							
Age (years)		58.28 (15.66)	44.40 (14.14)	0.95 (0.91, 0.99)	**0.013**		
Gender	male	71 (67.6%)	1 (10%)	1 (Ref)			
	female	34 (32.7%)	9 (90.0%)	18.53 (2.25, 152.26)	**0.007**		
Citizenship	Swiss	74 (71.2%)	3 (30.0%)	1 (Ref)		1 (Ref)	
	Non-Swiss	30 (28.8%)	7 (70.0%)	5.76 (1.39, 23.75)	**0.016**	9.83 (1.62, 59.64)	**0.013**
Cultural background	Central/Western Europe	86 (82.7%)	5 (50.0%)	1 (Ref)		1 (Ref)	
	Other	18 (17.3%)	5 (50.0%)	4.78 (1.25, 18.24)	**0.022**	15.05 (1.3, 174.21)	**0.030**
Religious affiliation	Christian	61 (58.7%)	5 (50.0%)	1 (Ref)		1 (Ref)	
	Non-Christian religion	10 (9.6%)	4 (40.0%)	4.88 (1.12, 21.33)	**0.035**	8.62 (0.75, 99.52)	0.084
	No religious affiliation	33 (31.7%)	1 (10.0%)	0.37 (0.04, 3.3)	0.373	0.24 (0.02, 2.68)	0.249
Civil status	Married/Partnership	69 (66.3%)	5 (50.0%)	1 (Ref)		1 (Ref)	
	Widowed/separated/single	35 (33.7%)	5 (50.0%)	1.97 (0.53, 7.27)	0.308	0.67 (0.14, 3.31)	0.621
Children	no	30 (29.7%)	3 (30%)	1 (Ref)		1 (Ref)	
	yes	71 (70.3%)	7 (70.0%)	1 (0.24, 4.13)	1.000	3.36 (0.47, 24)	0.226
Current job situation	Employed	53 (54%)	8 (80%)	1 (Ref)		1 (Ref)	
	Not employed	46 (46%)	2 (20%)	0.29 (0.06, 1.43)	0.127	0.56 (0.08, 4.04)	0.567
**Illness-related factors**							
Timepoint of COVID-19 diagnosis^a^, mean (SD)		31.27 (12.02)	26.50 (11.49)	0.97 (0.91, 1.02)	0.229	1.03 (0.95, 1.12)	0.497
Duration of hospitalization (days), mean (SD)		9.14 (6.51)	6.40 (3.98)	0.89 (0.75, 1.06)	0.204	0.9 (0.73, 1.12)	0.337
Severity of illness (NEWS score), mean (SD)		6.31 (3.76)	5.20 (3.26)	0.92 (0.77, 1.1)	0.370	1.1 (0.86, 1.41)	0.454
Comorbidity (CCI), mean (SD)		2.40 (2.17)	0.90 (1.20)	0.61 (0.38, 1)	0.050	0.76 (0.33, 1.76)	0.528
Self-perceived overall health status (Euroqol), mean (SD)		75.2 (16.39)	64.1 (18.95)	0.96 (0.93, 1)	0.053	0.97 (0.93, 1.02)	0.261
Antibiotics during hospitalization	no	70 (67.3%)	8 (80%)	1 (Ref)		1 (Ref)	
	yes	34 (32.7%)	2 (20.0%)	0.51 (0.1, 2.56)	0.417	0.7 (0.11, 4.6)	0.707
Investigational therapy	no	29 (27.9%)	5 (50%)	1 (Ref)		1 (Ref)	
	yes	75 (72.1%)	5 (50.0%)	0.39 (0.1, 1.44)	0.156	0.43 (0.08, 2.35)	0.328
Anxiolytics during hospitalization	no	88 (85.4%)	6 (60%)	1 (Ref)		1 (Ref)	
	yes	15 (14.6%)	4 (40.0%)	3.91 (0.99, 15.52)	0.052	3.18 (0.56, 17.97)	0.190
ICU stay	no	89 (84.8%)	8 (80%)	1 (Ref)		1 (Ref)	
	yes	16 (15.2%)	2 (20.0%)	1.39 (0.27, 7.16)	0.693	2.5 (0.31, 19.82)	0.387
Intubation	no	95 (90.5%)	9 (90%)	1 (Ref)		1 (Ref)	
	yes	10 (9.5%)	1 (10.0%)	1.06 (0.12, 9.21)	0.961	1.14 (0.07, 17.82)	0.926
**Psychosocial factors**							
Pre-existing psychological comorbidities	no	90 (87.4%)	7 (70%)	1 (Ref)		1 (Ref)	
	yes	13 (12.6%)	3 (30.0%)	2.97 (0.68, 12.93)	0.148	1.11 (0.2, 6.03)	0.906
Psychotropic drugs	no	94 (90.4%)	8 (80%)	1 (Ref)		1 (Ref)	
	yes	10 (9.6%)	2 (20.0%)	2.35 (0.44, 12.62)	0.319	1.37 (0.18, 10.37)	0.761
Resilience (CD-RISC), mean (SD)		32.08 (5.53)	26.00 (9.26)	0.88 (0.79, 0.97)	**0.010**	0.94 (0.82, 1.07)	0.360
Perceived Stress (PSS), mean (SD)		21.10 (6.73)	34.50 (6.16)	1.3 (1.08, 1.57)	**0.005**	78.64 (0.04, 160746.13)	0.262
Self-perceived stigmatization (VAS 0–10), mean (SD)		2.58 (3.16)	3.50 (3.33)	1.09 (0.85, 1.39)	0.492	1.07 (0.82, 1.4)	0.597
Consumption of COVID-19 media reports	no	17 (16.8%)	2 (20%)	1 (Ref)		1 (Ref)	
	yes	84 (83.2%)	8 (80.0%)	0.81 (0.16, 4.15)	0.800	1.42 (0.21, 9.83)	0.720
Duration of COVID-19 media consumption mean (SD)		36.29 (31.38)	41.88 (29.75)	1.01 (0.98, 1.03)	0.627	1.02 (0.99, 1.05)	0.231
Worries due to COVID-19 media reports, mean (SD)		3.77 (3.06)	7.00 (3.16)	1.4 (1.09, 1.81)	**0.008**	1.36 (1.02, 1.82)	**0.039**
Frequency of contacts with relatives	Daily	88 (86.3%)	9 (90.0%)	1 (Ref)		1 (Ref)	
	Less than daily	14 (13.7%)	1 (10.0%)	0.7 (0.08, 5.95)	0.743	1.13 (0.11, 11.79)	0.916
Type of communication between patients and relatives	Telephone, text and other	59 (57.8%)	6 (60.0%)	1 (Ref)		1 (Ref)	
	Video calls and visits	43 (42.2%)	4 (40.0%)	0.91 (0.24, 3.44)	0.895	0.38 (0.06, 2.52)	0.318
*Current worries and burden (VAS 0–10)*							
Worries about uncertain prognosis, mean (SD)		5.52 (3.15)	8.30 (1.95)	1.56 (1.08, 2.23)	**0.017**	1.41 (0.95, 2.09)	0.091
Burden of isolation measures, mean (SD)		4.74 (3.61)	7.40 (3.17)	1.27 (1.01, 1.58)	**0.039**	1.11 (0.86, 1.43)	0.439
Burden of boredom, mean (SD)		2.87 (3.29)	4.00 (3.77)	1.1 (0.91, 1.33)	0.333	0.9 (0.68, 1.19)	0.466
Worries about health of relatives, mean (SD)		4.87 (3.59)	6.90 (3.51)	1.19 (0.97, 1.47)	0.102	1.08 (0.84, 1.41)	0.539
Burden of missing relatives, mean (SD)		5.07 (3.67)	7.80 (3.01)	1.3 (1.01, 1.68)	**0.041**	1.14 (0.87, 1.48)	0.345
Worries about job situation, mean (SD)		1.37 (2.81)	1.60 (3.37)	1.03 (0.83, 1.28)	0.807	0.81 (0.6, 1.1)	0.184
Worries about finances, mean (SD)		0.93 (2.34)	1.80 (3.01)	1.13 (0.91, 1.4)	0.285	1.07 (0.82, 1.39)	0.616
Worries about medical care, mean (SD)		0.62 (1.59)	0	n.a.	n.a.	n.a.	n.a.
Other worries, mean (SD)		1.93 (3.60)	0.80 (2.53)	0.88 (0.68, 1.15)	0.350	0.91 (0.68, 1.22)	0.518
*Helpfulness of coping strategies (VAS 0–10)*							
Social contacts, mean (SD)		7.65 (2.64)	8.11 (1.83)	1.08 (0.8, 1.45)	0.610	1.03 (0.72, 1.47)	0.865
Distraction, mean (SD)		5.59 (3.52)	4.50 (3.67)	0.92 (0.73, 1.16)	0.466	0.84 (0.62, 1.14)	0.271
Tranquilizers, mean (SD)		0.44 (1.86)	3.33 (5.77)	1.31 (0.97, 1.76)	0.073	n.a.	n.a.
Other, mean (SD)		5.97 (4.22)	6.00 (4.00)	1 (0.79, 1.28)	0.989	0.94 (0.67, 1.32)	0.720
**Hospital-related factors (VAS 0–10)**							
Involvement of psychosocial care team	no	92 (88.5%)	8 (80%)	1 (Ref)		1 (Ref)	
	yes	12 (11.5%)	2 (20.0%)	1.92 (0.36, 10.1)	0.443	0.77 (0.09, 6.49)	0.806
Contradicting information given by medical team, mean (SD)		0.88 (1.85)	2.60 (3.03)	1.33 (1.04, 1.69)	**0.022**	1.06 (0.78, 1.44)	0.715
Perceived competence of treating physician, mean (SD)		8.79 (1.41)	8.50 (1.84)	0.88 (0.57, 1.34)	0.543	1.07 (0.67, 1.72)	0.774
Burden of having no visitors, mean (SD)		3.53 (3.32)	6.70 (3.37)	1.32 (1.07, 1.64)	**0.010**	1.18 (0.93, 1.49)	0.179
Missing physical closeness (VAS 0–10), mean (SD)		4.61 (3.69)	6.70 (3.62)	1.18 (0.97, 1.44)	0.101	1.07 (0.87, 1.32)	0.514
**b.**							
		**Multivariate overall model**					
		**OR (95%CI)**	***p***				
**Sociodemographic factors**							
Citizenship	Swiss	1 (Ref)					
	Non-Swiss	4.24 (0.78, 23.08)	0.095				
Cultural background	Central/Western Europe	1 (Ref)					
	Other	1.38 (0.25, 7.50)	0.38				
**Psychosocial factors**							
Worries due to COVID-19 media reports, mean (SD)		1.40 (1.08, 1.81)	**0.010**				

Data presented as n (%) or mean (standard deviation). Abbreviations: SD, standard deviation; OR, Odds Ratio; 95% CI, 95% Confidence Interval; COVID-19, Coronavirus disease 2019; NEWS, National Early Warning Score; CCI, Charlson Comorbidity Index; ICU, Intensive Care Unit; COVID-19, Coronavirus disease 2019; CD-RISC, Connor-Davidson Resilience Scale; PSS, Perceived Stress Scale; VAS, Visual Analogue Scale.

^a^consecutive days, starting with day 0 for first patients hospitalized

Only three relatives (2%) showed clinically relevant symptoms of PTSD. Due to the low number of events, no regression models were calculated.

## Discussion

In this Swiss prospective observational cohort study assessing the prevalence of psychological distress and potentially associated factors among COVID-19 patients and their relatives after hospital discharge, we found considerable rates of psychological distress in both groups which are higher than those among the Swiss general population in 2017 [77] as well as those of a large sample of the Swiss general population during the COVID-19 pandemic [21, 22]. Importantly, several associated factors were identified and some of these psychosocial and isolation-related factors seem to be addressable during routine hospital care and might be at least partially modifiable. Several points of our analysis deserve further comment.

First, the prevalence of psychological distress in our patient sample is in line with the results from Wu et al. [78] and Zhang et al. [79], who were among the first to evaluate psychological outcome in Chinese COVID-19 patients. Wu et al found 14% and 11% of patients to show at least mild symptoms [78], whereas Zhang et al found 21% and 29% [79] to show at least moderate symptoms of anxiety and depression, respectively. Bo et al. [33] found in an observational study which included 714 patients in China that almost all (96.2%) reported symptoms of PTSD during hospitalization. It must be noted that these symptoms do not reflect a PTSD diagnosis and findings may therefore not be interpreted as the rate of PTSD in this sample. The lower rate of patients with high PTSD symptom levels in our sample may be explained by the later time point at which patients were assessed. The higher rate reported by Bo et al. [33] may thus reflect symptoms of acute stress due to COVID-19 and isolation remitting within one month [80]. This is in line with symptoms of acute stress disorder remitting within one month after a traumatic event, and only a minority of patients developing full PTSD [80]. Our study reveals that relatives of COVID-19 patients might be affected to a similar extent, with 22.9% showing psychological distress and 16.3% and 15% showing symptoms of anxiety and depression, respectively. Studies evaluating the general population during the current pandemic found considerably high and increased levels of psychological distress [18, 19, 21–23, 25, 30, 31, 81–83], potentially related to environmental factors such as quarantine [12, 84], socioeconomic effects, and the risk of infection. However, several longitudinal studies did not find an increase in psychological distress in the general population before and during the first months of the pandemic [27–29]. First studies further differentiating between individuals who have a relative with COVID-19 and those who do not, suggest that having a sick relative causes significantly higher levels of distress [46.7 vs. 27.7%; 79, 84, 85]. These individuals might therefore require increased clinical attention tailored to their needs in order to prevent adverse long-term psychological burden [85–87].

Second, we identified several factors associated with psychological distress. Regarding gender disparities in the general population, woman are twice as likely to develop psychological sequelae [88]. In line with this and the findings of Wu et al. [89], female patients were more likely than males to report increased levels of psychological distress. Interestingly, this association was present for patients but not for relatives. This is in contrast to previous literature focusing on relatives of critically ill patients, in which being female was considered an important risk factor for psychological burden in relatives [58, 90, 91]. However, these studies focused on relatives of patients hospitalized in the ICU for a variety of reasons not related to COVID-19, and outcomes were measured three months after ICU discharge, which may limit the comparability with our specific population of relatives having a loved one hospitalized for COVID-19 [91]. Research on relatives of ICU patients has also shown that the likelihood of a high psychological burden was up to 18 times higher in relatives who felt that they were given incomplete information regarding their loved one [45, 58] or in relatives whose loved one died in the ICU [58, 92, 93]. This is in line with our finding that relatives of COVID-19 patients had more psychological distress and depression if the patient had died. The effect of patient outcomes on family members’ psychological burden is still a controversial topic in the literature, with some studies reporting no association between patient death and relatives’ psychological outcome, such as PTSD [45, 94]. However, a recent Dutch study found that people bereaved due to COVID-19 appear to have higher levels of prolonged grief disorder as well as persistent complex bereavement disorder compared to natural bereavement but not unnatural bereavement [95]. COVID-19 may be considered an unnatural and unexpected type of death which could explain the increased levels of distress in bereaved relatives in our study potentially leading to an increase in grief disorders during the COVID-19 pandemic.

Interestingly, while previous research has shown that patients with serious illnesses or hospitalization in the ICU are at increased risk of developing psychological sequelae [49, 96, 97], such associations were not found in our sample. In fact, apart from death of the patient, other illness-related factors such as comorbidity, severity of illness, ICU stay or mechanical ventilation were not associated with patients’ or relatives’ psychological outcomes. Psychological distress, however, was associated with subjective overall health in both patients and relatives, emphasizing the significance of considering an individual’s self-perception of their current health.

Further, relatives who were not employed were more likely to experience psychological distress than working relatives as expected based on general knowledge of the negative impact of unemployment on mental health across populations. In line with this, Shi et al. [84] identified employment as a potential protective factor in family members suffering from anxiety or depression in a large sample of the Chinese general population during the pandemic.

Among psychosocial factors, access to media coverage of COVID-19 was found to be a potential risk factor in prior studies [98], and concerns have been raised by leading mental health experts [47, 51, 99]. Previous research has shown that viewing media coverage of mass trauma may increase long-term distress [100, 101]. Hence, in persons at risk of distress, reducing media overconsumption might be beneficial.

Also, patients who reported higher perceived stress (i.e., experienced increased levels of perceived helplessness and lower levels of self-efficacy) and relatives who reported higher overall burden due to COVID-19 were more likely to show psychological distress. Patients who had daily contact with relatives or received support from personal social networks and patients with higher levels of resilience appeared to have lower levels of psychological distress.

Interestingly, frequency of contact with relatives showed a strong association with psychological distress and is potentially modifiable. While quarantine measures normally do not allow modifications, regular interaction with relatives might act as a protective factor in the development of psychological distress. Such interactions could also be done using new technology including face-to-face interactions over the smartphone or other devices. Current research into effective interventions to reduce depression and anxiety suggests that physical exercise is a potentially effective coping strategy and could be used during a lockdown [102–105].

Third, for both patients and relatives, resilience emerged as the most relevant factor associated with psychological distress and high PTSD symptom levels according to the DSM. However, both variables were assessed simultaneously and thus no causal conclusions can be made. Resilience may be defined as a person’s emotional and mental capacity to adapt well when experiencing critical life events [106–108]. With regard to resilience during the COVID-19 pandemic, leading mental health experts emphasize the need for access to mental health support [109–112] and the World Health Organization recently published specific recommendations [113, 114]. The latter are divided in several sections which are addressed to the general population, health care workers and team leaders, caretakers and people in isolation, respectively. They include short information and psychoeducation elements as well as specific recommendations and coping strategies, adapted to the current pandemic. Future research should evaluate whether interventions targeting core factors of resilience such as coping through social support and facilitating higher perceived self-efficacy are able to reduce the negative psychological impact of COVID-19.

Finally, we are aware of some limitations. As this is an observational study it is only hypotheses generating. Further, due to language barriers, death and restricted accessibility, we could not include all consecutive patients and relatives, potentially inducing a selection bias. Therefore, our data need confirmation in a larger cohort of patients and relatives. Due to the clinical circumstances of COVID-19 and patients’ hospitalization such as isolation measures and the sudden and rapidly increasing number of cases in early March 2020, it was neither feasible to assess patients nor all relatives during patients’ hospitalization. We thus contacted patients and relatives at 30 days after discharge and asked for recalled information regarding baseline and follow-up, which could introduce recall bias.

## Conclusions

A considerable proportion of COVID-19 patients as well as their relatives show symptoms of psychological distress 30 days after hospital discharge. Several psychosocial and isolation-related factors such as resilience, perceived stress, frequency of contact with relatives and worries due to media reports were associated with adverse outcome and are at least partially modifiable. Along with previously known risk factors for psychological distress in hospitalized patients, our findings could be used to identify patients and relatives at increased risk of experiencing psychological distress over the long term, and to tailor interventions accordingly. Future research should assess whether interventions targeting these risk factors improve psychological outcome of COVID-19 patients and their relatives.

## Supporting information

S1 File (DOCX)

S1 Data (XLSX)

## References

[1] Guan WJ, Ni ZY, Hu Y, Liang WH, Ou CQ, He JX, et al. Clinical Characteristics of Coronavirus Disease 2019 in China. N Engl J Med. 2020. 10.1056/NEJMoa2002032 PMC7092819 32109013

[2] Lescure FX, Bouadma L, Nguyen D, Parisey M, Wicky PH, Behillil S, et al. Clinical and virological data of the first cases of COVID-19 in Europe: a case series. Lancet Infect Dis. 2020. 10.1016/S1473-3099(20)30200-0 32224310 PMC7156120

[3] Bhatraju PK, Ghassemieh BJ, Nichols M, Kim R, Jerome KR, Nalla AK, et al. Covid-19 in Critically Ill Patients in the Seattle Region—Case Series. N Engl J Med. 2020. 10.1056/NEJMoa2004500 32227758 PMC7143164

[4] Qiu H, Wu J, Hong L, Luo Y, Song Q, Chen D. Clinical and epidemiological features of 36 children with coronavirus disease 2019 (COVID-19) in Zhejiang, China: an observational cohort study. Lancet Infect Dis. 2020. 10.1016/S1473-3099(20)30198-5 PMC7158906 32220650

[5] Richardson S, Hirsch JS, Narasimhan M, Crawford JM, McGinn T, Davidson KW, et al. Presenting Characteristics, Comorbidities, and Outcomes Among 5700 Patients Hospitalized With COVID-19 in the New York City Area. JAMA. 2020. 10.1001/jama.2020.6775 PMC7177629 32320003

[6] Petrilli CM, Jones SA, Yang J, Rajagopalan H, O’Donnell L, Chernyak Y, et al. Factors associated with hospital admission and critical illness among 5279 people with coronavirus disease 2019 in New York City: prospective cohort study. BMJ. 2020;369:m1966. 10.1136/bmj.m1966 32444366 PMC7243801

[7] Armstrong RA, Kane AD, Cook TM. Outcomes from intensive care in patients with COVID-19: a systematic review and meta-analysis of observational studies. Anaesthesia. 2020. 10.1111/anae.15201 32602561

[8] Luo Y, Chua CR, Xiong Z, Ho RC, Ho CSH. A Systematic Review of the Impact of Viral Respiratory Epidemics on Mental Health: An Implication on the Coronavirus Disease 2019 Pandemic. Front Psychiatry. 2020;11:565098. 10.3389/fpsyt.2020.565098 33329106 PMC7719673

[9] Krishnamoorthy Y, Nagarajan R, Saya GK, Menon V. Prevalence of psychological morbidities among general population, healthcare workers and COVID-19 patients amidst the COVID-19 pandemic: A systematic review and meta-analysis. Psychiatry Research. 2020;293:113382. 10.1016/j.psychres.2020.113382 32829073 PMC7417292

[10] Wu T, Jia X, Shi H, Niu J, Yin X, Xie J, et al. Prevalence of mental health problems during the COVID-19 pandemic: A systematic review and meta-analysis. J Affect Disord. 2020;281:91–8. 10.1016/j.jad.2020.11.117 33310451 PMC7710473

[11] Rogers JP, Chesney E, Oliver D, Pollak TA, McGuire P, Fusar-Poli P, et al. Psychiatric and neuropsychiatric presentations associated with severe coronavirus infections: a systematic review and meta-analysis with comparison to the COVID-19 pandemic. The Lancet Psychiatry. 2020;7(7):611–27. 10.1016/S2215-0366(20)30203-0 32437679 PMC7234781

[12] Brooks SK, Webster RK, Smith LE, Woodland L, Wessely S, Greenberg N, et al. The psychological impact of quarantine and how to reduce it: rapid review of the evidence. Lancet. 2020;395(10227):912–20. 10.1016/S0140-6736(20)30460-8 32112714 PMC7158942

[13] Xiang Y-T, Yang Y, Li W, Zhang L, Zhang Q, Cheung T, et al. Timely mental health care for the 2019 novel coronavirus outbreak is urgently needed. The Lancet Psychiatry. 2020;7(3):228–9. 10.1016/S2215-0366(20)30046-8 32032543 PMC7128153

[14] Maunder R. Stress, coping and lessons learned from the SARS outbreak. Hosp Q. 2003;6(4):49–50, 4. 10.12927/hcq..16480 14628529

[15] Rubin GJ, Wessely S. The psychological effects of quarantining a city. BMJ. 2020;368:m313. 10.1136/bmj.m313 31992552

[16] Tsang HW, Scudds RJ, Chan EY. Psychosocial impact of SARS. Emerg Infect Dis. 2004;10(7):1326–7. 10.3201/eid1007.040090 15338536 PMC3323309

[17] Zhu Y, Zhang L, Zhou X, Li C, Yang D. The impact of social distancing during COVID-19: A conditional process model of negative emotions, alienation, affective disorders, and post-traumatic stress disorder. J Affect Disord. 2020;281:131–7. 10.1016/j.jad.2020.12.004 33316718 PMC7723399

[18] Gloster AT, Lamnisos D, Lubenko J, Presti G, Squatrito V, Constantinou M, et al. Impact of COVID-19 pandemic on mental health: An international study. PLoS One. 2020;15(12):e0244809. 10.1371/journal.pone.0244809 33382859 PMC7774914

[19] Pierce M, Hope H, Ford T, Hatch S, Hotopf M, John A, et al. Mental health before and during the COVID-19 pandemic: a longitudinal probability sample survey of the UK population. The Lancet Psychiatry. 2020;7(10):883–92. 10.1016/S2215-0366(20)30308-4 32707037 PMC7373389

[20] McGinty EE, Presskreischer R, Han H, Barry CL. Psychological Distress and Loneliness Reported by US Adults in 2018 and April 2020. JAMA. 2020;324(1):93–4. 10.1001/jama.2020.9740 32492088 PMC7270868

[21] de Quervain D, Aerni A, Amini E, Bentz D, Coynel D, Gerhards C, et al. The Swiss Corona Stress Study. in press. 2020.

[22] De Quervain D, Aerni A, E. A, Bentz D, Coynel D, Gerhards C, et al. The Swiss Corona Stress Study: second pandemic wave, November 2020. [Journal Article]. In press 2020.

[23] Bäuerle A, Steinbach J, Schweda A, Beckord J, Hetkamp M, Weismüller B, et al. Mental Health Burden of the COVID-19 Outbreak in Germany: Predictors of Mental Health Impairment. J Prim Care Community Health. 2020;11:2150132720953682. 10.1177/2150132720953682 32865107 PMC7457643

[24] Twenge JM, Joiner TE. U.S. Census Bureau-assessed prevalence of anxiety and depressive symptoms in 2019 and during the 2020 COVID-19 pandemic. Depress Anxiety. 2020;37(10):954–6. 10.1002/da.23077 32667081 PMC7405486

[25] Daly M, Sutin AR, Robinson E. Depression reported by US adults in 2017–2018 and March and April 2020. J Affect Disord. 2021;278:131–5. 10.1016/j.jad.2020.09.065 32956962 PMC7490280

[26] van der Velden PG, Contino C, Das M, van Loon P, Bosmans MWG. Anxiety and depression symptoms, and lack of emotional support among the general population before and during the COVID-19 pandemic. A prospective national study on prevalence and risk factors. J Affect Disord. 2020;277:540–8. 10.1016/j.jad.2020.08.026 32889378 PMC7438386

[27] van der Velden PG, Hyland P, Contino C, von Gaudecker HM, Muffels R, Das M. Anxiety and depression symptoms, the recovery from symptoms, and loneliness before and after the COVID-19 outbreak among the general population: Findings from a Dutch population-based longitudinal study. PLoS One. 2021;16(1):e0245057. 10.1371/journal.pone.0245057 33411843 PMC7790276

[28] van Tilburg TG, Steinmetz S, Stolte E, van der Roest H, de Vries DH. Loneliness and mental health during the COVID-19 pandemic: A study among Dutch older adults. J Gerontol B Psychol Sci Soc Sci. 2020. 10.1093/geronb/gbaa111 32756931 PMC7454922

[29] Breslau J, Finucane ML, Locker AR, Baird MD, Roth EA, Collins RL. A longitudinal study of psychological distress in the United States before and during the COVID-19 pandemic. Prev Med. 2021;143:106362. 10.1016/j.ypmed.2020.106362 33388325 PMC9753596

[30] Vindegaard N, Benros ME. COVID-19 pandemic and mental health consequences: Systematic review of the current evidence. Brain Behav Immun. 2020. 10.1016/j.bbi.2020.05.048 32485289 PMC7260522

[31] Cooke JE, Eirich R, Racine N, Madigan S. Prevalence of posttraumatic and general psychological stress during COVID-19: A rapid review and meta-analysis. Psychiatry Res. 2020;292:113347. 10.1016/j.psychres.2020.113347 32763477 PMC7392847

[32] Dorman-Ilan S, Hertz-Palmor N, Brand-Gothelf A, Hasson-Ohayon I, Matalon N, Gross R, et al. Anxiety and Depression Symptoms in COVID-19 Isolated Patients and in Their Relatives. Front Psychiatry. 2020;11:581598. 10.3389/fpsyt.2020.581598 33192727 PMC7591814

[33] Mazza MG, De Lorenzo R, Conte C, Poletti S, Vai B, Bollettini I, et al. Anxiety and depression in COVID-19 survivors: Role of inflammatory and clinical predictors. Brain Behav Immun. 2020. 10.1016/j.bbi.2020.07.037 32738287 PMC7390748

[34] Tomasoni D, Bai F, Castoldi R, Barbanotti D, Falcinella C, Mule G, et al. Anxiety and depression symptoms after virological clearance of COVID-19: A cross-sectional study in Milan, Italy. J Med Virol. 2021;93(2):1175–9. 10.1002/jmv.26459 32841387 PMC7461061

[35] Liu D, Baumeister RF, Veilleux JC, Chen C, Liu W, Yue Y, et al. Risk factors associated with mental illness in hospital discharged patients infected with COVID-19 in Wuhan, China. Psychiatry Res. 2020;292:113297. 10.1016/j.psychres.2020.113297 32707218 PMC7355324

[36] Poyraz BC, Poyraz CA, Olgun Y, Gurel O, Alkan S, Ozdemir YE, et al. Psychiatric morbidity and protracted symptoms after COVID-19. Psychiatry Res. 2021;295:113604. 10.1016/j.psychres.2020.113604 33296818 PMC7695976

[37] Huang C, Huang L, Wang Y, Li X, Ren L, Gu X, et al. 6-month consequences of COVID-19 in patients discharged from hospital: a cohort study. The Lancet. 2021. 10.1016/S0140-6736(20)32656-8 33428867 PMC7833295

[38] Paz C, Mascialino G, Adana-Diaz L, Rodriguez-Lorenzana A, Simbana-Rivera K, Gomez-Barreno L, et al. Behavioral and sociodemographic predictors of anxiety and depression in patients under epidemiological surveillance for COVID-19 in Ecuador. PLoS One. 2020;15(9):e0240008. 10.1371/journal.pone.0240008 32997705 PMC7526886

[39] Prout TA, Zilcha-Mano S, Aafjes-van Doorn K, Bekes V, Christman-Cohen I, Whistler K, et al. Identifying Predictors of Psychological Distress During COVID-19: A Machine Learning Approach. Front Psychol. 2020;11:586202. 10.3389/fpsyg.2020.586202 33240178 PMC7682196

[40] Şahan E, Ünal SM, Kırpınar İ. Can we predict who will be more anxious and depressed in the COVID-19 ward? Journal of Psychosomatic Research. 2021;140. 10.1016/j.jpsychores.2020.110302 PMC7683951 33264750

[41] Bonsaksen T, Heir T, Schou-Bredal I, Ekeberg O, Skogstad L, Grimholt TK. Post-Traumatic Stress Disorder and Associated Factors during the Early Stage of the COVID-19 Pandemic in Norway. Int J Environ Res Public Health. 2020;17(24). 10.3390/ijerph17249210 33317135 PMC7764050

[42] Pfefferbaum B, Newman E, Nelson SD, Nitiema P, Pfefferbaum RL, Rahman A. Disaster media coverage and psychological outcomes: descriptive findings in the extant research. Curr Psychiatry Rep. 2014;16(9):464. 10.1007/s11920-014-0464-x 25064691 PMC4144190

[43] Guest R, Tran Y, Gopinath B, Cameron ID, Craig A. Prevalence and psychometric screening for the detection of major depressive disorder and post-traumatic stress disorder in adults injured in a motor vehicle crash who are engaged in compensation. BMC Psychol. 2018;6(1):4. 10.1186/s40359-018-0216-5 29467035 PMC5822643

[44] Rosman L, Sico JJ, Lampert R, Gaffey AE, Ramsey CM, Dziura J, et al. Posttraumatic Stress Disorder and Risk for Stroke in Young and Middle-Aged Adults: A 13-Year Cohort Study. Stroke. 2019;50(11):2996–3003. 10.1161/STROKEAHA.119.026854 31619151

[45] Zimmerli M, Tisljar K, Balestra GM, Langewitz W, Marsch S, Hunziker S. Prevalence and risk factors for post-traumatic stress disorder in relatives of out-of-hospital cardiac arrest patients. Resuscitation. 2014;85(6):801–8. 10.1016/j.resuscitation.2014.02.022 24598377

[46] Metzger K, Gamp M, Tondorf T, Hochstrasser S, Becker C, Luescher T, et al. Depression and anxiety in relatives of out-of-hospital cardiac arrest patients: Results of a prospective observational study. J Crit Care. 2019;51:57–63. 10.1016/j.jcrc.2019.01.026 30745287

[47] Fiorillo A, Gorwood P. The consequences of the COVID-19 pandemic on mental health and implications for clinical practice. Eur Psychiatry. 2020;63(1):e32. 10.1192/j.eurpsy.2020.35 32234102 PMC7156565

[48] Hosey MM, Needham DM. Survivorship after COVID-19 ICU stay. Nat Rev Dis Primers. 2020;6(1):60. 10.1038/s41572-020-0201-1 32669623 PMC7362322

[49] Tingey JL, Bentley JA, Hosey MM. COVID-19: Understanding and mitigating trauma in ICU survivors. Psychol Trauma. 2020;12(S1):S100–S4. 10.1037/tra0000884 32584106

[50] Parker C, Shalev D, Hsu I, Shenoy A, Cheung S, Nash S, et al. Depression, Anxiety, and Acute Stress Disorder Among Patients Hospitalized With Coronavirus Disease 2019: A Prospective Cohort Study. Psychosomatics. 2020. 10.1016/j.psym.2020.10.001 PMC7546958 33198962

[51] Holmes EA, O’Connor RC, Perry VH, Tracey I, Wessely S, Arseneault L, et al. Multidisciplinary research priorities for the COVID-19 pandemic: a call for action for mental health science. The Lancet Psychiatry. 2020. 10.1016/S2215-0366(20)30168-1 PMC7159850 32304649

[52] Hu T, Zhang D, Wang J. A meta-analysis of the trait resilience and mental health. Personality and Individual Differences. 2015;76:18–27.

[53] Blanc J, Briggs AQ, Seixas AA, Reid M, Jean-Louis G, Pandi-Perumal SR. Addressing psychological resilience during the coronavirus disease 2019 pandemic: a rapid review. Curr Opin Psychiatry. 2021;34(1):29–35. 10.1097/YCO.0000000000000665 33230041 PMC7751836

[54] van der Meulen E, van der Velden PG, van Aert RCM, van Veldhoven M. Longitudinal associations of psychological resilience with mental health and functioning among military personnel: A meta-analysis of prospective studies. Soc Sci Med. 2020;255:112814. 10.1016/j.socscimed.2020.112814 32388075

[55] Scali J, Gandubert C, Ritchie K, Soulier M, Ancelin M-L, Chaudieu I. Measuring Resilience in Adult Women Using the 10-Items Connor-Davidson Resilience Scale (CD-RISC). Role of Trauma Exposure and Anxiety Disorders. PLOS ONE. 2012;7(6):e39879. 10.1371/journal.pone.0039879 22768152 PMC3387225

[56] van der Meulen E, van Veldhoven MJPM, van der Velden PG. Stability of psychological resilience of police officers: A three-wave latent class analysis. Personality and Individual Differences. 2019;144:120–4.

[57] von Elm E, Altman DG, Egger M, Pocock SJ, Gotzsche PC, Vandenbroucke JP, et al. The Strengthening the Reporting of Observational Studies in Epidemiology (STROBE) statement: guidelines for reporting observational studies. Lancet. 2007;370(9596):1453–7. 10.1016/S0140-6736(07)61602-X 18064739

[58] Azoulay E, Pochard F, Kentish-Barnes N, Chevret S, Aboab J, Adrie C, et al. Risk of post-traumatic stress symptoms in family members of intensive care unit patients. Am J Respir Crit Care Med. 2005;171(9):987–94. 10.1164/rccm.200409-1295OC 15665319

[59] Charlson ME, Pompei P, Ales KL, MacKenzie CR. A new method of classifying prognostic comorbidity in longitudinal studies: development and validation. J Chronic Dis. 1987;40(5):373–83. 10.1016/0021-9681(87)90171-8 3558716

[60] Smith GB, Prytherch DR, Meredith P, Schmidt PE, Featherstone PI. The ability of the National Early Warning Score (NEWS) to discriminate patients at risk of early cardiac arrest, unanticipated intensive care unit admission, and death. Resuscitation. 2013;84(4):465–70. 10.1016/j.resuscitation.2012.12.016 23295778

[61] Dolan P. Modeling valuations for EuroQol health states. Med Care. 1997;35(11):1095–108. 10.1097/00005650-199711000-00002 9366889

[62] EuroQol G. EuroQol—a new facility for the measurement of health-related quality of life. Health Policy. 1990;16(3):199–208. 10.1016/0168-8510(90)90421-9 10109801

[63] Taylor JM. Psychometric analysis of the Ten-Item Perceived Stress Scale. Psychol Assess. 2015;27(1):90–101. 10.1037/a0038100 25346996

[64] Cohen S, Kamarck T, Mermelstein R. A Global Measure of Perceived Stress. Journal of Health and Social Behavior. 1983;24(4):385–96. 6668417

[65] Connor KM, Davidson JR. Development of a new resilience scale: the Connor-Davidson Resilience Scale (CD-RISC). Depress Anxiety. 2003;18(2):76–82. 10.1002/da.10113 12964174

[66] Zager Kocjan G, Kavcic T, Avsec A. Resilience matters: Explaining the association between personality and psychological functioning during the COVID-19 pandemic. Int J Clin Health Psychol. 2021;21(1):100198. 10.1016/j.ijchp.2020.08.002 33363581 PMC7753029

[67] Rainey EE, Petrey LB, Reynolds M, Agtarap S, Warren AM. Psychological factors predicting outcome after traumatic injury: the role of resilience. The American Journal of Surgery. 2014;208(4):517–23. 10.1016/j.amjsurg.2014.05.016 25124293

[68] Sarubin N, Gutt D, Giegling I, Bühner M, Hilbert S, Krähenmann O, et al. Erste Analyse der psychometrischen Eigenschaften und Struktur der deutschsprachigen 10-und 25-Item Version der Connor-Davidson Resilience Scale (CD-RISC). Zeitschrift für Gesundheitspsychologie. 2015.

[69] Davidson JRT. Connor-Davidson Resilience Scale (CD-RISC) Manual. 2018.

[70] Zigmond AS, Snaith RP. The hospital anxiety and depression scale. Acta Psychiatr Scand. 1983;67(6):361–70. 10.1111/j.1600-0447.1983.tb09716.x 6880820

[71] Bjelland I, Dahl AA, Haug TT, Neckelmann D. The validity of the Hospital Anxiety and Depression Scale. An updated literature review. J Psychosom Res. 2002;52(2):69–77. 10.1016/s0022-3999(01)00296-3 11832252

[72] Maercker A, Schützwohl M. Erfassung von psychischen Belastungsfolgen: Die Impact of Event Skala-revidierte Version (IES-R). Diagnostica. 1998(44(3)):130–41.

[73] Horowitz M, Wilner N, Alvarez W. Impact of Event Scale: a measure of subjective stress. Psychosom Med. 1979;41(3):209–18. 10.1097/00006842-197905000-00004 472086

[74] Weiss DS. The impact of event scale: revised. Cross-cultural assessment of psychological trauma and PTSD: Springer; 2007. p. 219–38.

[75] Creamer M, Bell R, Failla S. Psychometric properties of the impact of event scale—revised. Behaviour research and therapy. 2003;41(12):1489–96. 10.1016/j.brat.2003.07.010 14705607

[76] Sterne JA, White IR, Carlin JB, Spratt M, Royston P, Kenward MG, et al. Multiple imputation for missing data in epidemiological and clinical research: potential and pitfalls. Bmj. 2009;338. 10.1136/bmj.b2393 19564179 PMC2714692

[77] Schuler D, Tuch A, Peter C. Psychische Gesundheit in der Schweiz. Monitoring 2020. Schweizerisches Gesundheitsobservatorium (Obsan); 2020.

[78] Wu C, Hu X, Song J, Yang D, Xu J, Cheng K, et al. Mental health status and related influencing factors of COVID-19 survivors in Wuhan, China. Clin Transl Med. 2020. 10.1002/ctm2.52 32508037 PMC7300592

[79] Zhang J, Lu H, Zeng H, Zhang S, Du Q, Jiang T, et al. The differential psychological distress of populations affected by the COVID-19 pandemic. Brain Behav Immun. 2020;87:49–50. 10.1016/j.bbi.2020.04.031 32304883 PMC7156946

[80] American Psychiatric Association. Diagnostic and statistical manual of mental disorders (DSM-5®): American Psychiatric Pub; 2013.

[81] Huang Y, Zhao N. Generalized anxiety disorder, depressive symptoms and sleep quality during COVID-19 outbreak in China: a web-based cross-sectional survey. Psychiatry Res. 2020;288:112954. 10.1016/j.psychres.2020.112954 32325383 PMC7152913

[82] Tian F, Li H, Tian S, Yang J, Shao J, Tian C. Psychological symptoms of ordinary Chinese citizens based on SCL-90 during the level I emergency response to COVID-19. Psychiatry Res. 2020;288:112992. 10.1016/j.psychres.2020.112992 32302816 PMC7151383

[83] Wang C, Pan R, Wan X, Tan Y, Xu L, Ho CS, et al. Immediate Psychological Responses and Associated Factors during the Initial Stage of the 2019 Coronavirus Disease (COVID-19) Epidemic among the General Population in China. Int J Environ Res Public Health. 2020;17(5). 10.3390/ijerph17051729 32155789 PMC7084952

[84] Shi L, Lu ZA, Que JY, Huang XL, Liu L, Ran MS, et al. Prevalence of and Risk Factors Associated With Mental Health Symptoms Among the General Population in China During the Coronavirus Disease 2019 Pandemic. JAMA Netw Open. 2020;3(7):e2014053. 10.1001/jamanetworkopen.2020.14053 32609353 PMC7330717

[85] Tanoue Y, Nomura S, Yoneoka D, Kawashima T, Eguchi A, Shi S, et al. Mental health of family, friends, and co-workers of COVID-19 patients in Japan. Psychiatry Res. 2020;291:113067. 10.1016/j.psychres.2020.113067 32535504 PMC7202826

[86] Azoulay E, Kentish-Barnes N. A 5-point strategy for improved connection with relatives of critically ill patients with COVID-19. The Lancet Respiratory Medicine. 2020;8(6). 10.1016/S2213-2600(20)30223-X 32380024 PMC7198186

[87] Singer J, Spiegel JA, Papa A. Preloss grief in family members of COVID-19 patients: Recommendations for clinicians and researchers. Psychol Trauma. 2020. 10.1037/tra0000876 32478543

[88] World Health Organization. Gender disparities in mental health. Geneva: World Health Organization, DEPENDENCE DOMHAS; 2001.

[89] Wu C, Hu X, Song J, Du C, Song Y, Yang D, et al. Mental Health Status of Survivors Following COVID-19 in Wuhan, China: A Descriptive Study. 2020.

[90] Pochard F, Azoulay E, Chevret S, Lemaire F, Hubert P, Canoui P, et al. Symptoms of anxiety and depression in family members of intensive care unit patients: ethical hypothesis regarding decision-making capacity. Crit Care Med. 2001;29(10):1893–7. 10.1097/00003246-200110000-00007 11588447

[91] Gil-Julia B, Bernat-Adell MD, Collado-Boira EJ, Moles Julio MP, Ballester-Arnal R. Psychological distress in relatives of critically ill patients: Risk and protective factors. J Health Psychol. 2018:1359105318817357. 10.1177/1359105318817357 30582372

[92] Lautrette A, Darmon M, Megarbane B, Joly LM, Chevret S, Adrie C, et al. A communication strategy and brochure for relatives of patients dying in the ICU. N Engl J Med. 2007;356(5):469–78. 10.1056/NEJMoa063446 17267907

[93] Gries CJ, Engelberg RA, Kross EK, Zatzick D, Nielsen EL, Downey L, et al. Predictors of symptoms of posttraumatic stress and depression in family members after patient death in the ICU. Chest. 2010;137(2):280–7. 10.1378/chest.09-1291 19762549 PMC2816640

[94] Anderson WG, Arnold RM, Angus DC, Bryce CL. Posttraumatic stress and complicated grief in family members of patients in the intensive care unit. Journal of general internal medicine. 2008;23(11):1871–6. 10.1007/s11606-008-0770-2 18780129 PMC2585673

[95] Eisma MC, Tamminga A, Smid GE, Boelen PA. Acute grief after deaths due to COVID-19, natural causes and unnatural causes: An empirical comparison. J Affect Disord. 2021;278:54–6. 10.1016/j.jad.2020.09.049 32950843 PMC7487144

[96] Hatch R, Young D, Barber V, Griffiths J, Harrison DA, Watkinson P. Anxiety, Depression and Post Traumatic Stress Disorder after critical illness: a UK-wide prospective cohort study. Crit Care. 2018;22(1):310. 10.1186/s13054-018-2223-6 30466485 PMC6251214

[97] Jackson JC, Pandharipande PP, Girard TD, Brummel NE, Thompson JL, Hughes CG, et al. Depression, post-traumatic stress disorder, and functional disability in survivors of critical illness in the BRAIN-ICU study: a longitudinal cohort study. Lancet Respir Med. 2014;2(5):369–79. 10.1016/S2213-2600(14)70051-7 24815803 PMC4107313

[98] Dubey S, Biswas P, Ghosh R, Chatterjee S, Dubey MJ, Chatterjee S, et al. Psychosocial impact of COVID-19. Diabetes Metab Syndr. 2020;14(5):779–88. 10.1016/j.dsx.2020.05.035 PMC7255207 32526627

[99] Amsalem D, Dixon LB, Neria Y. The Coronavirus Disease 2019 (COVID-19) Outbreak and Mental Health: Current Risks and Recommended Actions. JAMA Psychiatry. 2020. 10.1001/jamapsychiatry.2020.1730 32579160

[100] Neria Y, Sullivan GM. Understanding the mental health effects of indirect exposure to mass trauma through the media. JAMA. 2011;306(12):1374–5. 10.1001/jama.2011.1358 21903818 PMC3637659

[101] Thompson RR, Jones NM, Holman EA, Silver RC. Media exposure to mass violence events can fuel a cycle of distress. Sci Adv. 2019;5(4):eaav3502. 10.1126/sciadv.aav3502 31001584 PMC6469939

[102] Cooney GM, Dwan K, Greig CA, Lawlor DA, Rimer J, Waugh FR, et al. Exercise for depression. Cochrane Database of Systematic Reviews. 2013(9). 10.1002/14651858.CD004366.pub6 24026850 PMC9721454

[103] Stubbs B, Vancampfort D, Hallgren M, Firth J, Veronese N, Solmi M, et al. EPA guidance on physical activity as a treatment for severe mental illness: a meta-review of the evidence and Position Statement from the European Psychiatric Association (EPA), supported by the International Organization of Physical Therapists in Mental Health (IOPTMH). European Psychiatry. 2018;54:124–44. 10.1016/j.eurpsy.2018.07.004 30257806

[104] Ensari I, Greenlee TA, Motl RW, Petruzzello SJ. META-ANALYSIS OF ACUTE EXERCISE EFFECTS ON STATE ANXIETY: AN UPDATE OF RANDOMIZED CONTROLLED TRIALS OVER THE PAST 25 YEARS. Depression and Anxiety. 2015;32(8):624–34. 10.1002/da.22370 25899389

[105] Rethorst CD. Exercise for Depression/Anxiety. The Encyclopedia of Clinical Psychology 2015. p. 1–5.

[106] Southwick SM, Charney DS. The science of resilience: implications for the prevention and treatment of depression. Science. 2012;338(6103):79–82. 10.1126/science.1222942 23042887

[107] Russo SJ, Murrough JW, Han M-H, Charney DS, Nestler EJ. Neurobiology of resilience. Nature Neuroscience. 2012;15(11):1475–84. 10.1038/nn.3234 23064380 PMC3580862

[108] Holz NE, Tost H, Meyer-Lindenberg A. Resilience and the brain: a key role for regulatory circuits linked to social stress and support. Mol Psychiatry. 2020;25(2):379–96. 10.1038/s41380-019-0551-9 31628419

[109] Killgore WDS, Taylor EC, Cloonan SA, Dailey NS. Psychological resilience during the COVID-19 lockdown. Psychiatry Res. 2020;291:113216. 10.1016/j.psychres.2020.113216 32544705 PMC7280133

[110] Lupe SE, Keefer L, Szigethy E. Gaining resilience and reducing stress in the age of COVID-19. Curr Opin Gastroenterol. 2020;36(4):295–303. 10.1097/MOG.0000000000000646 32398567

[111] Carvalho PMM, Moreira MM, de Oliveira MNA, Landim JMM, Neto MLR. The psychiatric impact of the novel coronavirus outbreak. Psychiatry Res. 2020;286:112902. 10.1016/j.psychres.2020.112902 32146248 PMC7133679

[112] Duan L, Zhu G. Psychological interventions for people affected by the COVID-19 epidemic. The Lancet Psychiatry. 2020;7(4):300–2. 10.1016/S2215-0366(20)30073-0 32085840 PMC7128328

[113] Ghebreyesus TA. Addressing mental health needs: an integral part of COVID‐19 response. World Psychiatry. 2020;19(2):129. 10.1002/wps.20768 32394569 PMC7214944

[114] World Health Organization. Mental health and psychosocial considerations during the COVID-19 outbreak, 18 March 2020. World Health Organization; 2020.

