# Supplementary material for: Prevalence and factors associated with psychological burden in COVID-19 patients and their relatives: A prospective observational cohort study
Source: PLoS One. 2021 May 5;16(5):e0250590. doi: 10.1371/journal.pone.0250590 (PMC8099094; doi:10.1371/journal.pone.0250590)
Supplement: S1 File — (DOCX) [file pone.0250590.s001.docx]

**S1 File.**

STROBE Statement—Prevalence and risk factors for psychosocial burden in COVID-19 patients and their relatives: A prospective observational cohort study

|  | | **Item No.** | **Recommendation** | | | **Page  No.** | **Relevant text from manuscript** |  |
| --- | --- | --- | --- | --- | --- | --- | --- | --- |
| **Title and abstract** | | 1 | (*a*) Indicate the study’s design with a commonly used term in the title or the abstract | | | 1, 2 | A prospective observational cohort study |  |
|  |  |  | (*b*) Provide in the abstract an informative and balanced summary of what was done and what was found | | | 2, 3 |  |  |
| **Introduction** | | | | | | |  |  |
| Background/rationale | | 2 | Explain the scientific background and rationale for the investigation being reported | | | 4-9 |  |  |
| Objectives | | 3 | State specific objectives, including any prespecified hypotheses | | | 9 | To assess in parallel the prevalence and potential risk and protective factors associated with psychological distress in COVID-19 patients and their relatives. |  |
| **Methods** | | | | | | |  |  |
| Study design | | 4 | Present key elements of study design early in the paper | | | 9 | Prospective observational cohort study |  |
| Setting | | 5 | Describe the setting, locations, and relevant dates, including periods of recruitment, exposure, follow-up, and data collection | | | 9 | Two participating tertiary care hospitals (University Hospital Basel and the Kantonsspital Aarau, Switzerland) from March until June 2020 |  |
| Participants | | 6 | (*a*) *Cohort study*—Give the eligibility criteria, and the sources and methods of selection of participants. Describe methods of follow-up | | | 9-10 | We screened all consecutively admitted COVID-19 patients and their closest relatives upon hospitalization. Relatives were chosen according to surrogate decision-making rank (spouse > parents/adult children > others) as indicated in patients’ medical records. We contacted relatives during hospitalization and patients about one month after hospital discharge by phone and invited them to participate in our study. |  |
|  |  |  | (*b*) *Cohort study*—For matched studies, give matching criteria and number of exposed and unexposed | | | n.a. |  |  |
| Variables | | 7 | Clearly define all outcomes, exposures, predictors, potential confounders, and effect modifiers. Give diagnostic criteria, if applicable | | | 10-15 | Paragraphs “Collection of potential predictor and outcome variables baseline and follow-up data of patients and relatives” and “Outcomes” |  |
| Data sources/ measurement | | 8* | For each variable of interest, give sources of data and details of methods of assessment (measurement). Describe comparability of assessment methods if there is more than one group | | | 10-15 |  |  |
| Bias | | 9 | Describe any efforts to address potential sources of bias | | |  | To minimize inter-observer variability in conducting the interview, a thorough training for all interviewers was conducted by the study coordinators. |  |
| Study size | | 10 | Explain how the study size was arrived at | | | 17 | Between March and June 2020, a total of 301 patients with COVID-19 were hospitalized in the University Hospital Basel (n=198) and the Kantonsspital Aarau (n=103) (S1 Figure 1). Forty of these patients (13.3%) died during hospitalization or within 30 days after discharge, 54 (17.9%) were unable to speak the local language (German), 32 (16.6%) met exclusion criteria such as dementia or severe underlying psychiatric conditions, 29 (9.6%) were not reachable by phone for assessment , and 20 (6.6%) did not give informed consent. In 12 (4%) of all 301 hospitalized patients no relatives were documented in the medical charts. Of the 298 remaining relatives, 15.4% did not speak German and 7% were excluded due to other criteria, e.g., cognitive impairment or being already included in the patient sample. Forty-five (15.1%) were not reachable by phone and 24 (8.1%) did not give informed consent. Thus, the final cohort consisted of 126 patients and 153 relatives. |  |
| Quantitative variables | 11 | | | Explain how quantitative variables were handled in the analyses. If applicable, describe which groupings were chosen and why | | 15-17 | We conducted all analyses separately for each the patient and the relative sample. We evaluated associations to explore independent associations between potential predictors and outcomes, separately in two steps, through univariate and multivariate analyses. |  |
| Statistical methods | 12 | | | (*a*) Describe all statistical methods, including those used to control for confounding | | 15-17 | We further investigated the associations between each predictor variable and the respective outcome by adjusting each of these analyses for age, gender and study center. In a next step, we calculated multivariable logistic regression models first for each factor adjusted for age, gender and study center, second within the four domains. |  |
|  |  |  |  | (*b*) Describe any methods used to examine subgroups and interactions | | n.a. |  |  |
|  |  |  |  | (*c*) Explain how missing data were addressed | | 15-16 | To account for missing data in predictors used in the multivariate analysis, we imputed datasets using multiple imputations by chained equations. Imputations were calculated using multiple covariables within domains also including main outcomes to reduce bias as previously suggested. (…) Model performance of imputed data was also compared to those of crude values to check consistency (data not shown). We found a similar pattern when doing a full set analysis (see table S1 and S2 in the Supplementary Material). |  |
|  |  |  |  | (*d*) *Cohort study*—If applicable, explain how loss to follow-up was addressed | | n.a. |  |  |
|  |  |  |  | (*e*) Describe any sensitivity analyses | | 17 | Areas under the curve (AUC) were calculated to evaluate the prognostic value of the potential predictors for outcome. |  |
| **Results** | | | | | | | |  |
| Participants | 13* | | | (a) Report numbers of individuals at each stage of study—eg numbers potentially eligible, examined for eligibility, confirmed eligible, included in the study, completing follow-up, and analysed | | 17, Figure 1 | Between March and May 2020, 301 patients with COVID-19 were hospitalized in the University Hospital of Basel (n=198) and the Kantonsspital Aarau (n=103). 40 died, 54 were unable to speak the local language, 32 had dementia or severe underlying psychiatric conditions, 29 were not reachable by phone for assessment and did not give informed consent. Thus, the final cohort consisted of 126 patients and 153 relatives. |  |
|  |  |  |  | (b) Give reasons for non-participation at each stage | | 17, Figure 1 | See 13(a) |  |
|  |  |  |  | (c) Consider use of a flow diagram | | Figure 1 |  |  |
| Descriptive data | 14* | | | (a) Give characteristics of study participants (eg demographic, clinical, social) and information on exposures and potential confounders | | Table 1 |  |  |
|  |  |  |  | (b) Indicate number of participants with missing data for each variable of interest | |  |  |  |
|  |  |  |  | (c) *Cohort study*—Summarise follow-up time (eg, average and total amount) | | n.a. |  |  |
| Outcome data | 15* | | | *Cohort study*—Report numbers of outcome events or summary measures over time | | 17 |  |  |
|  |  |  |  | *Case-control study—*Report numbers in each exposure category, or summary measures of exposure | | *n.a.* |  |  |
|  |  |  |  | *Cross-sectional study—*Report numbers of outcome events or summary measures | | *n.a.* |  |  |
| Main results | 16 | | | (*a*) Give unadjusted estimates and, if applicable, confounder-adjusted estimates and their precision (eg, 95% confidence interval). Make clear which confounders were adjusted for and why they were included | | 19-31 Table 2, 3 |  |  |
|  |  |  |  | (*b*) Report category boundaries when continuous variables were categorized | |  |  |  |
|  |  |  |  | (*c*) If relevant, consider translating estimates of relative risk into absolute risk for a meaningful time period | | n.a. |  |  |
| Other analyses | 17 | | | | Report other analyses done—eg analyses of subgroups and interactions, and sensitivity analyses | 31-41, Table 4a+b S1 and S2 Table |  | |
| **Discussion** | | | | | | | |  |
| Key results | 18 | | | | Summarise key results with reference to study objectives | 36-40 | In this Swiss prospective observational cohort studies assessing the prevalence of psychological distress and potentially associated factors among COVID-19 patients and their relatives after hospital discharge, we found considerable rates of psychological distress in both groups which are higher than those among the Swiss general population in 2017 [77] as well as those of a large sample of the Swiss general population during the COVID-19 pandemic [21, 22]. Importantly, several associated factors were identified and some of these psychosocial and isolation-related factors seem to be addressable during routine hospital care and might be at least partially modifiable. | |
| Limitations | 19 | | | | Discuss limitations of the study, taking into account sources of potential bias or imprecision. Discuss both direction and magnitude of any potential bias | 40-41 | Finally, we are aware of some limitations. As this is an observational study it is only hypotheses generating. Further, due to language barriers, death and restricted accessibility, we could not include all consecutive patients and relatives, potentially inducing a selection bias. Therefore, our data need confirmation in a larger cohort of patients and relatives. Due to the clinical circumstances of COVID-19 and patients’ hospitalization such as isolation measures and the sudden and rapidly increasing number of cases in early March 2020, it was neither feasible to assess patients nor all relatives during patients’ hospitalization. We thus contacted patients and relatives at 30 days after discharge and asked for recalled information regarding baseline and follow-up, which could introduce recall bias. | |
| Interpretation | 20 | | | | Give a cautious overall interpretation of results considering objectives, limitations, multiplicity of analyses, results from similar studies, and other relevant evidence | 41 | A considerable proportion of COVID-19 patients as well as their relatives show symptoms of psychological distress 30 days after hospital discharge. Several psychosocial and isolation-related factors such as resilience, perceived stress, frequency of contact with relatives and worries due to media reports were associated with adverse outcome and are at least partially modifiable. Along with previously known risk factors for psychological distress in hospitalized patients, our findings could be used to identify patients and relatives at increased risk of experiencing psychological distress over the long term, and to tailor interventions accordingly. Future research should assess whether interventions targeting these risk factors improve psychological outcome of COVID-19 patients and their relatives. | |
| Generalisability | 21 | | | | Discuss the generalisability (external validity) of the study results | 40-41 | See also limitations above | |
| **Other information** | | | | | **13** | | |  |
| Funding | 22 | | | | Give the source of funding and the role of the funders for the present study and, if applicable, for the original study on which the present article is based | 15 | SH and study team were funded by the Swiss National Foundation (SNF) (Ref 10001C_192850/1 and 10531C_182422) | |

**Table S1.** Factors associated with psychological distress in patients – raw data without imputed values

|  |  | Overall multivariate model | |
| --- | --- | --- | --- |
|  |  | **OR (95%CI)** | ***p*** |
| Sociodemographic factors |  |  |  |
| Age (years) |  |  |  |
| Gender | male | 1 (Ref) |  |
|  | female | 1.65 (0.22, 12.21) | 0.625 |
| Religious affiliation | Christian | 1 (Ref) |  |
|  | Non-Christian religion | 1.51 (0.03, 88.06) | 0.843 |
|  | No religious affiliation | 1.98 (0.22, 18.14) | 0.547 |
| Current job situation | Employed | 1 (Ref) |  |
|  | Not employed | 2.83 (0.44, 18.31) | 0.274 |
| Illness-related factors |  |  |  |
| Self-perceived overall health status (Euroqol), mean (SD) |  | 0.98 (0.92, 1.04) | 0.497 |
| Psychosocial factors |  |  |  |
| Resilience (CD-RISC), mean (SD) |  | 0.79 (0.65, 0.96) | **0.018** |
| Perceived Stress (PSS), mean (SD) |  | 1.12 (0.97, 1.30) | 0.121 |
| Frequency of contacts with relatives | Daily | 1 (Ref) |  |
|  | Less than daily | 3.08 (0.04, 258.56) | 0.618 |
| *Helpfulness of coping strategies (VAS 0-10)* |  |  |  |
| Social contacts, mean (SD) |  | 0.89 (0.63, 1.23) | 0.472 |

Data are presented as n (%) or mean (standard deviation)

Abbreviations: SD, standard deviation; OR, odds ratio; 95%CI, 95% Confidence Interval; COVID-19, Coronavirus disease 2019; CD-RISC, Connor-Davidson Resilience Scale; PSS, Perceived Stress Scale; VAS, visual analogue scale

**Table S2.** Factors associated with psychological distress in relatives – raw data without imputed values

|  |  | Overall multivariate model | |
| --- | --- | --- | --- |
|  |  | **OR (95% CI)** | ***p*** |
| Sociodemographic factors |  |  |  |
| Children | no | 1 (Ref) |  |
|  | yes | 3.16 (0.60, 16.49) | 0.173 |
| Current job situation | Employed | 1 (Ref) |  |
|  | Not employed | 2.56 (0.75, 8.80) | 0.135 |
| Illness-related factors |  |  |  |
| Self-perceived overall health status (Euroqol), mean (SD) |  | 0.99 (0.95, 1.03) | 0.617 |
| Death of patient | no | 1 (Ref) |  |
|  | yes | 1.39 (0.26, 7.29) | 0.701 |
| Psychosocial factors |  |  |  |
| Resilience (CD-RISC), mean (SD) |  | 0.87 (0.77, 0.99) | **0.030** |
| *Current worries and burdens (VAS 0-10)* |  |  |  |
| Perceived overall burden due to COVID-19, mean (SD) |  | 1.59 (1.18, 2.15) | **0.003** |
| *Helpfulness of coping strategies (VAS 0-10)* |  |  |  |
| Sports, mean (SD) |  | 0.87 (0.75, 1.02) | 0.088 |

Data presented as n (%) or mean (standard deviation)

Abbreviations: SD, standard deviation; OR, Odds Ratio; 95% CI, 95% Confidence Interval; COVID-19, Coronavirus disease 2019; CD-RISC, Connor-Davidson Resilience Scale
